# Association of Health Insurance, Geography, and Race and Ethnicity With Disparities in Receipt of Recommended Postpartum Care in the US

**DOI:** 10.1001/jamahealthforum.2022.3292

**Published:** 2022-10-14

**Authors:** Julia D. Interrante, Lindsay K. Admon, Caitlin Carroll, Carrie Henning-Smith, Phoebe Chastain, Katy B. Kozhimannil

**Affiliations:** 1 University of Minnesota Rural Health Research Center, University of Minnesota School of Public Health, Minneapolis; 2 Division of Health Policy and Management, University of Minnesota School of Public Health, Minneapolis; 3 Department of Obstetrics and Gynecology, University of Michigan, Ann Arbor; 4 Institute for Healthcare Policy and Innovation, University of Michigan, Ann Arbor

## Abstract

**Importance:**

Little is known about the quality of postpartum care or disparities in the content of postpartum care associated with health insurance, rural or urban residency, and race and ethnicity.

**Objectives:**

To examine receipt of recommended postpartum care content and to describe variations across health insurance type, rural or urban residence, and race and ethnicity.

**Design, Settings, and Participants:**

This cross-sectional survey of patients with births from 2016 to 2019 used data from the Pregnancy Risk Assessment Monitoring System (43 states and 2 jurisdictions). A population-based sample of patients conducted by state and local health departments in partnership with the Centers for Disease Control and Prevention were surveyed about maternal experiences 2 to 6 months after childbirth (mean weighted response rate, 59.9%). Patients who attended a postpartum visit were assessed for content at that visit. Analyses were performed November 2021 to July 2022.

**Exposures:**

Medicaid or private health insurance, rural or urban residence, and race and ethnicity (non-Hispanic White or racially minoritized groups).

**Main Outcomes and Measures:**

Receipt of 2 postpartum care components recommended by national quality standards (depression screening and contraceptive counseling), and/or other recommended components (smoking screening, abuse screening, birth spacing counseling, eating and exercise discussions) with estimated risk-adjusted predicted probabilities and percentage-point (pp) differences.

**Results:**

Among the 138 073 patient-respondents, most (59.5%) were in the age group from 25 to 34 years old; 59 726 (weighted percentage, 40%) were insured by Medicaid; 27 721 (15%) were rural residents; 9718 (6%) were Asian, 24 735 (15%) were Black, 22 210 (15%) were Hispanic, 66 323 (60%) were White, and fewer than 1% were Indigenous (Native American/Alaska Native) individuals. Receipt of both depression screening and contraceptive counseling both significantly lower for Medicaid-insured patients (1.2 pp lower than private; 95% CI, –2.1 to –0.3), rural residents (1.3 pp lower than urban; 95% CI, –2.2 to –0.4), and people of racially minoritized groups (0.8 pp lower than White individuals; 95% CI, –1.6 to –0.1). The highest receipt of these components was among privately insured White urban residents (80%; 95% CI, 79% to 81%); the lowest was among privately insured racially minoritized rural residents (75%; 95% CI, 72% to 78%). Receipt of all other components was significantly higher for Medicaid-insured patients (6.1 pp; 95% CI, 5.2 to 7.0), rural residents (1.1 pp; 95% CI, 0.1 to 2.0), and people of racially minoritized groups (8.5 pp; 95% CI, 7.7 to 9.4). The highest receipt of these components was among Medicaid-insured racially minoritized urban residents (34%; 95% CI, 33% to 35%), the lowest was among privately insured White urban residents (19%; 95% CI, 18% to 19%).

**Conclusions and Relevance:**

The findings of this cross-sectional survey of postpartum individuals in the US suggest that inequities in postpartum care content were extensive and compounded for patients with multiple disadvantaged identities. Examining only 1 dimension of identity may understate the extent of disparities. Future studies should consider the content of postpartum care visits.

## Key Points

**Question:**

Does receipt and content of recommended postpartum care differ across health insurance type, rural or urban residence, and race and ethnicity?

**Findings:**

In this cross-sectional survey of 138 073 patients who attended a postpartum visit, statistically significant self-reported differences in the content of postpartum care were found across health insurance type, rural or urban residency, and race and ethnicity. Intersectional differences (eg, insurance by geography) were consistently larger than differences across individual categories (eg, insurance alone).

**Meaning:**

The findings of this population-based survey study suggest that inequities in the content of postpartum care received are extensive across patients’ insurance type, rural or urban residence, and racial and ethnic identities, and these disparities are compounded for patients with multiple intersecting disadvantaged identities.

## Teaser

This cross-sectional survey study of more than 100 000 individuals examines how health insurance, geography, and race and ethnicity may be associated with disparities in the content of postpartum care visits.

## Introduction

In the US, three-quarters of all birthing people report health challenges during the first year after childbirth, including breastfeeding difficulties, fatigue, pain, and depression and/or anxiety.^1^ Managing these problems is especially complicated for the 1 of 3 postpartum patients with chronic health conditions.^2^ In the US, postpartum care is often limited to 1 visit, which is insufficient and not attended by many patients.^3,4^ Half of postpartum patients report not receiving all of the care they wanted and 30% report feeling rushed.^1,5^ The potential consequences of inadequate postpartum care and treatment are substantial.^4,6^

Maternal health disparities reflect structural inequities across key aspects of patient sociodemographic identity: race and ethnicity, rural or urban geographic residence, and health insurance type. Poor maternal health outcomes are more common for Medicaid insured than for privately insured patients, for rural more so than urban residents, and people of racially minoritized groups than for non-Hispanic White patients.^7,8,9,10^ Systemic racism, discrimination, immigration policy, rural obstetric unit closures, and a lack of culturally and linguistically appropriate care impede access and are associated with poor perinatal outcomes for many patients of racially minoritized groups.^11,12^ For patients with multiple disadvantaged identities (ie, groups that are marginalized through experiences of discrimination and exclusion because of unequal power relationships), inequities in health and access to care may be compounded. Intersectionality of risk produces some of the highest risks for poor maternal outcomes among Black and Indigenous people who are rural residents.^13,14^ These same groups are overrepresented among Medicaid beneficiaries, compounding maternal health risks.^15,16^

There is little understanding of whether postpartum patients receive necessary care.^17^ Depression screening and contraceptive counseling are 2 components of postpartum care that are recommended by existing national quality standards and are often tied to financial incentives for clinicians, health care facilities, and insurers.^17,18,19,20^ The American College of Obstetricians and Gynecologists (ACOG) also recommend postpartum care include screening for tobacco use and counseling on relapse risks; follow-up on preexisting mental health concerns, including screening for abuse; discussions about birth spacing; and guidance on physical activity and healthy weight following pregnancy.^4^

Few studies have examined postpartum care beyond binary measures of receiving any vs no care.^3,17^ Even less is known about variation in the quality and content of postpartum care across patient identities, largely because of a lack of data collection in the postpartum period as well as a paucity of research during this period that disaggregates along intersectional lines.^17,21^ This study examines receipt of recommended components of postpartum care and describes variation in care across insurance type, rural or urban residence, and race and ethnicity, as well as the intersections of these patient identities.

## Methods

This analysis used deidentified data and was therefore exempted from review by the institutional review board at the study site. The study followed the Strengthening the Reporting of Observational Studies in Epidemiology (STROBE) reporting guideline.

### Study Design and Population

Data came from the Pregnancy Risk Assessment Monitoring System (PRAMS), a cross-sectional survey of maternal experiences conducted by state and local health departments in partnership with the US Centers for Disease Control and Prevention (CDC).^22^ We used PRAMS data from phase 8 surveys, which samples postpartum patients from birth certificate records in 45 sites with live births from 2016 to 2019 surveyed between 2 and 6 months after childbirth.^23^ For each survey year, data are released only from sites that meet a minimum response rate threshold (ie, 55% in 2016-2017 and 50% in 2018-2019), and as such, data availability and sample sizes vary by site-year as described in eTable 1 in Supplement 1.^23^

### Study Outcomes

**Figure 1.**
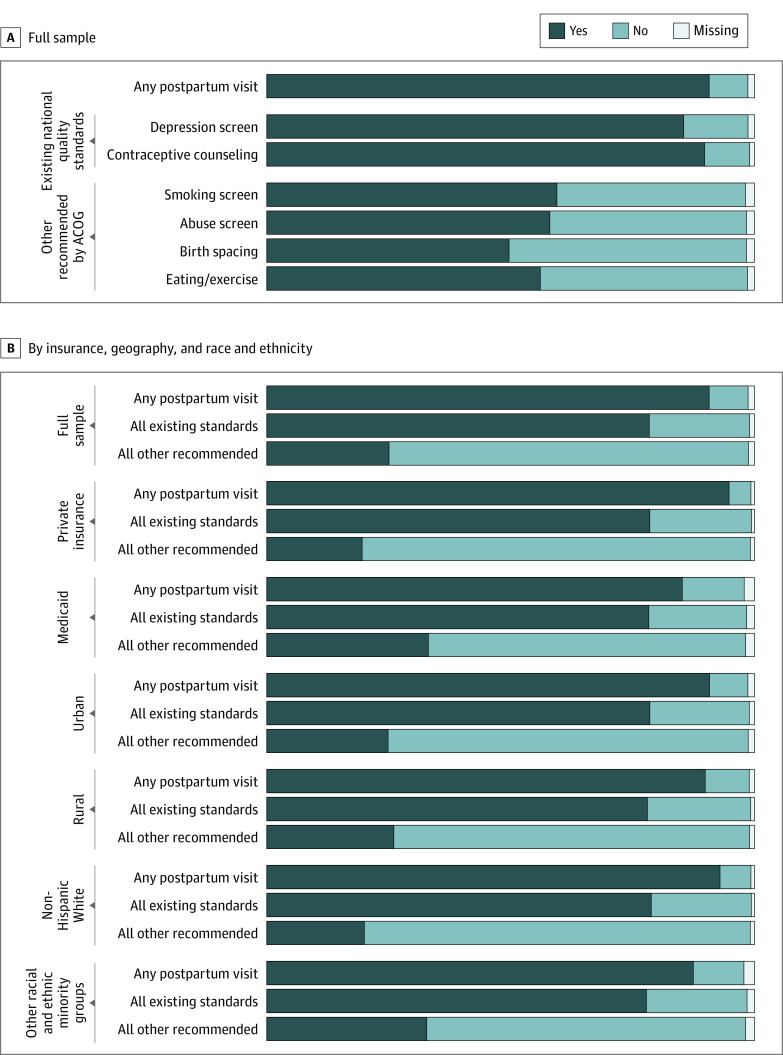
Weighted Percentage of Patients Who Received Guideline-Based Postpartum Care, by Health Insurance Type, Geography (Rural or Urban), and Race and Ethnicity A, Proportions across the full study sample. B, Proportions by health insurance, geography, and race and ethnicity. Data are PRAMS 2016 to 2019 (n = 153 683) and are weighted to account for sample design, nonresponse, and noncoverage. Percentages within specific care components are restricted to those patients attending a postpartum visit. ACOG refers to the American College of Obstetricians and Gynecologists, and PRAMS, Pregnancy Risk Assessment Monitoring System.

Outcomes were self-reported receipt of recommended postpartum care components during a postpartum visit. Survey respondents were asked whether they had attended a postpartum visit (defined in PRAMS as “the regular checkup a woman has about 4-6 weeks after she gives birth”) or had a home visit after birth “conducted by a doctor, nurse, or other health care worker.” If the patient had had a visit, PRAMS asked whether the patient had received these care components: depression screening, contraceptive counseling, smoking screening, abuse screening, birth spacing counseling, and eating and exercise discussions (eTable 2 in Supplement 1). Each question had a yes or no response option. Contraceptive counseling was defined as discussion or receipt of contraception. Care components were examined by (1) receipt of the 2 components recommended by existing national quality standards (depression, contraceptive)^17,18,19,20^ and (2) receipt of all other components recommended by ACOG (smoking, abuse, birth spacing, eating/exercise).^4^ Rates of nonresponse ranged from 0.4% to 3.0% and are reported in Figure 1 and eTable 3 in Supplement 1.

### Patient Identities

We examined patient identity by the race and ethnicity, rural or urban geographic residence, and health insurance information obtained from the PRAMS-linked birth certificate data, as indicators of access and to examine groups often marginalized or stereotyped based on these intersecting identities. Payer was measured as the primary payer for the childbirth episode.^24^ We only included patients with either Medicaid or private health insurance coverage (97% of all births in the sample; 5911 patients were excluded). Geographic residence of each patient was determined to be either rural or urban, assessed at the county level per the 2013 National Center for Health Statistics Urban-Rural Classification Scheme−metropolitan statistical areas are considered urban, and nonmetropolitan (micropolitan/noncore) are rural.^25^ Patients’ race and ethnicity were examined as indicators for maternal health risks that may be associated with racism^26^; these data are usually self-reported and routinely entered into the birth certificate application by the birthing facility. The PRAMS data do not include information on country of origin or immigration status, but do include language, which is often used as a proxy for acculturation.^27^ We dichotomized race and ethnicity into 2 groups: non-Hispanic White and “all other races and ethnicities” (Asian/Pacific Islander, non-Hispanic Black, Hispanic [preferred language, English/Spanish], Indigenous [American Indian/Alaska Native], and of multiple or other races [other/multiple]). The intersections of patient identities were also examined to assess how having multiple “disadvantaged” identities may affect patient care. We used aggregated categories of race and ethnicity to assist in estimation and comparison; however, we recognize that using aggregated categories may misclassify or obscure outcomes for some individuals.^28^ For intersectional examination, race and ethnicity were dichotomized as White or people of racially minoritized groups for interpretation in the main text. Further disaggregation by race and ethnicity and by specific care component is shown in eFigure 2 in Supplement 1.

### Statistical Analysis

All analyses were survey weighted, accounting for variation in sampling rates, stratification schemes, and nonresponse across site-years.^29^ We calculated weighted percentages and 95% CIs to describe the study population. Among patients attending a postpartum visit, we used multivariate logistic regression to calculate predicted probabilities and percentage-point risk differences (RD) in receipt of postpartum care content across individual and intersectional dimensions of insurance type, rural or urban residence, and race and ethnicity, using a separate regression model for individual and intersectional analyses.

To risk-standardize, analyses were adjusted for patients’ sociodemographic and health characteristics selected a priori based on current literature^30,31,32^: age (<20 years, 20-24, 25-34, ≥35), educational attainment (<high school, high school, >high school), marital status (married, not married), parity (multiparous, not multiparous), and childbirth type (cesarean, vaginal)—obtained from linked birth certificate data. Other variables from the PRAMS questionnaire included pregnancy intention (intended, if the pregnancy was wanted at the time or earlier; unintended, if pregnancy was wanted later, never wanted, or unsure), prepregnancy obesity (body mass index [BMI], >29 calculated as weight [kg] divided by height [m^2^]), and prepregnancy or pregnancy smoking (from 3 months before), physical abuse (from 12 months before), depression (from 3 months before), diabetes (preexisting or gestational), and high blood pressure (eg, hypertension, preeclampsia, eclampsia). Among study participants, 0.4% (n = 560) were missing data on health insurance type, rural or urban residence, and race and ethnicity, and 4.3% (n = 9106) were missing data on any covariates previously mentioned. In line with prior analyses using these data, missing values were controlled using indicators in adjusted analyses.

Sensitivity analyses were conducted to examine how differential nonresponse affected outcomes, including complete case analysis and multiple imputation, as well as to assess robustness of findings to missing data across site-years, including site clustering, restricting to sites with all years available, and restricting to later years. Significance was assessed based on 95% CIs excluding the null and defined as *P* <.05 using 2-sided hypothesis tests. All analyses were conducted from November 2021 to July 2022 using Stata, version 17 (StataCorp LLC).

## Results

### Patient Characteristics

Of 153 683 total patients, 138 073 had attended a postpartum visit. Of these, most (59.5%) were in the age group from 25 to 34 years old; 59 726 (weighted, 40%) were insured by Medicaid; 27 721 (15%) were rural residents; 9718 (6%) were Asian, 24 735 (15%) were Black, 22 210 (15%) were Hispanic (preferred language, 9% English and 6% Spanish), 66 323 (60%) were White, and fewer than 1% were Indigenous individuals. The mean weighted PRAMS response rate was 59.9% for site-years included in this study. Postpartum visit attendance was lower (85% and 87%) among Medicaid-insured and racially minoritized patients, respectively (Table). Characteristics of patients by visit attendance are described in eTable 4 in Supplement 1. Differences in health characteristics examined by separate and intersectional patient identities are provided in the Table and eTable 5 in Supplement 1.

**Table.** Participant Characteristics by Health Insurance Type, Geography (Rural or Urban), and Race and Ethnicity, PRAMS 2016 to 2019^a^

Characteristic	% (95% CI)						
	All (n = 153 683)	Health insurance type		Geographic residence		Race and ethnicity	
		Private (n = 82 770)	Medicaid (n = 70 904)	Urban (n = 122 207)	Rural (n = 31 437)	Non-Hispanic White (n = 74 389)	All other (n = 78 782)
Postpartum visit	90.7 (90.4-90.9)	94.8 (94.6-95.0)	85.2 (84.8-85.7)	90.8 (90.6-91.1)	89.9 (89.3-90.4)	93.0 (92.7-93.2)	87.4 (87.0-87.8)
Mean proportion of care components received	61.0 (60.8-61.3)	60.2 (59.9-60.5)	62.1 (61.7-62.5)	61.0 (60.7-61.3)	61.2 (60.5-61.8)	59.3 (59.0-59.6)	63.5 (63.1-64.0)
Among patients with a postpartum visit, No.	138 073	78 339	59 726	110 319	27 721	69 176	68 461
**Patient identities**							
Health insurance							
Private	59.6 (59.2-59.9)	NA	NA	61.3 (60.9-61.7)	49.8 (48.8-50.8)	71.8 (71.3-72.3)	40.7 (40.1-41.3)
Medicaid	40.5 (40.1-40.9)	NA	NA	38.7 (38.3-39.1)	50.2 (49.2-51.2)	28.2 (27.7-28.7)	59.3 (58.7-59.9)
Geographic residence							
Urban	84.7 (84.5-85.0)	87.2 (86.9-87.6)	81.1 (80.6-81.6)	NA	NA	80.4 (79.9-80.7)	91.4 (91.1-91.7)
Rural	15.3 (15.0-15.5)	12.8 (12.4-13.1)	18.9 (18.4-19.4)	NA	NA	19.7 (19.3-20.1)	8.6 (8.3-8.9)
Race and ethnicity							
Non-Hispanic White	60.4 (60.0-60.7)	72.7 (72.3-73.2)	42.1 (41.5-42.8)	57.2 (56.8-57.6)	77.7 (77.0-78.5)	NA	NA
Other races and ethnicities	39.3 (38.9-39.6)	26.9 (26.4-27.3)	57.6 (56.9-58.2)	42.4 (42.0-42.8)	22.1 (21.4-22.9)	NA	NA
Asian or Pacific Islander	5.6 (5.4-5.7)	6.6 (6.4-6.9)	4.0 (3.8-4.3)	6.4 (6.2-6.6)	1.0 (0.8-1.1)	NA	14.2 (13.8-14.6)
Non-Hispanic Black	14.9 (14.6-15.1)	8.5 (8.3-8.8)	24.2 (23.6-24.7)	16.1 (15.8-16.4)	8.1 (7.5-8.7)	NA	37.7 (37.2-38.3)
Hispanic (English preferred)	9.2 (9.0-9.4)	7.0 (6.8-7.3)	12.4 (12.0-12.8)	9.8 (9.6-10.1)	5.6 (5.2-6.0)	NA	23.4 (22.9-23.9)
Hispanic (Spanish preferred)	6.0 (5.8-6.2)	1.8 (1.7-2.0)	12.1 (11.7-12.6)	6.6 (6.3-6.8)	2.8 (2.5-3.1)	NA	15.2 (14.7-15.7)
Indigenous	0.7 (0.7-0.7)	0.3 (0.3-0.3)	1.3 (1.2-1.3)	0.4 (0.4-0.4)	2.5 (2.3-2.6)	NA	1.8 (1.7-1.9)
Other/multiple^b^	3.1 (2.9-3.2)	2.6 (2.5-2.8)	3.7 (3.5-4.0)	3.2 (3.0-3.3)	2.5 (2.2-2.7)	NA	7.8 (7.4-8.1)
**Sociodemographics**							
Age, y							
<20	3.9 (3.7-4.0)	1.3 (1.1-1.4)	7.7 (7.4-8.1)	3.4 (3.3-3.6)	6.3 (5.8-6.8)	2.9 (2.8-3.1)	5.3 (5.0-5.6)
20-24	17.6 (17.3-17.9)	10.7 (10.4-11.1)	27.7 (27.1-28.3)	16.1 (15.8-16.5)	25.6 (24.7-26.6)	15.4 (15.0-15.8)	21.0 (20.5-21.6)
25-34	59.5 (59.1-59.9)	64.6 (64.1-65.1)	52.1 (51.4-52.7)	60.1 (59.6-60.5)	56.5 (55.5-57.5)	62.5 (62.0-63.0)	54.9 (54.3-55.6)
≥35	19.0 (18.7-19.4)	23.5 (23.0-23.9)	12.5 (12.1-13.0)	20.4 (20.0-20.7)	11.6 (11.0-12.2)	19.2 (18.8-19.6)	18.7 (18.2-19.2)
Education							
<High school	9.6 (9.3-9.8)	2.1 (1.9-2.2)	20.6 (20.1-21.2)	9.3 (9.0-9.5)	11.2 (10.6-11.9)	5.1 (4.9-5.4)	16.5 (16.0-16.9)
High school	23.2 (22.9-23.6)	12.8 (12.5-13.2)	38.5 (37.8-39.1)	21.8 (21.4-22.2)	31.0 (30.0-32.0)	19.6 (19.1-20.0)	28.9 (28.3-29.5)
>High school	66.4 (66.0-66.8)	84.4 (84.0-84.8)	39.9 (39.2-40.5)	68.0 (67.6-68.4)	57.4 (56.4-58.4)	74.9 (74.4-75.3)	53.7 (53.1-54.4)
Married	63.9 (63.5-64.3)	82.7 (82.3-83.2)	36.1 (35.5-36.7)	64.9 (64.5-65.4)	58.1 (57.1-59.1)	74.1 (73.6-74.6)	48.1 (47.4-48.7)
**Other health characteristics**							
Multiparous	60.0 (59.6-60.4)	56.1 (55.6-56.6)	65.8 (65.2-66.4)	59.5 (59.0-59.9)	62.9 (62.0-63.9)	58.6 (58.0-59.1)	62.3 (61.7-62.9)
Cesarean delivery	33.5 (33.2-33.9)	33.8 (33.3-34.3)	33.2 (32.5-33.8)	33.6 (33.2-34.0)	33.2 (32.2-34.1)	32.4 (31.9-32.9)	35.4 (34.8-36.0)
Unintended pregnancy	38.8 (38.4-39.2)	28.3 (27.8-28.8)	54.4 (53.7-55.0)	38.1 (37.7-38.6)	42.6 (41.6-43.6)	32.3 (31.8-32.8)	48.9 (48.3-49.5)
Prepregnancy obesity	29.9 (29.6-30.3)	26.4 (25.9-26.9)	35.1 (34.5-35.8)	28.8 (28.4-29.2)	36.3 (35.4-37.3)	27.8 (27.3-28.3)	33.3 (32.7-33.9)
Prepregnancy or pregnancy							
Smoking	16.3 (16.0-16.6)	10.1 (9.7-10.4)	25.6 (25.0-26.1)	14.6 (14.2-14.9)	26.2 (25.3-27.1)	19.3 (18.9-19.7)	11.8 (11.4-12.2)
Abuse	4.1 (3.9-4.2)	2.1 (2.0-2.3)	6.9 (6.6-7.3)	3.7 (3.6-3.9)	5.9 (5.4-6.4)	3.6 (3.4-3.8)	4.8 (4.5-5.0)
Depression	17.7 (17.4-18.0)	13.9 (13.6-14.3)	23.3 (22.8-23.9)	16.6 (16.3-16.9)	24.0 (23.1-24.8)	19.6 (19.2-20.0)	14.9 (14.4-15.3)
Diabetes	12.0 (11.7-12.2)	11.7 (11.3-12.0)	12.4 (12.0-12.8)	12.0 (11.7-12.3)	11.9 (11.2-12.5)	11.0 (10.6-11.3)	13.5 (13.1-13.9)
High blood pressure	16.3 (16.0-16.6)	15.5 (15.1-15.9)	17.4 (16.9-17.9)	15.9 (15.5-16.2)	18.5 (17.8-19.3)	16.4 (16.0-16.8)	16.1 (15.6-16.6)

Abbreviations: NA, not applicable; PRAMS, Pregnancy Risk Assessment Monitoring System.

^a^ Data are weighted to account for sample design, nonresponse, and noncoverage. Sum of column percentages may not equal 100% because of missing data.

^b^ Other/multiple category includes respondents who self-identified as either “other non-White or mixed race.”

### Care Components by Patient Identities

Among patients who had attended a postpartum visit, 78% reported receiving both of the 2 care components recommended by existing national quality standards (depression screening and contraception counseling); 85% reported receiving any depression screening (“independent” receipt) and 90%, any contraceptive counseling (Figure 1). However, only 25% of patients who had a postpartum visit received all other recommended care components (smoking, abuse, birth spacing, eating and exercise). Independent receipt of the other components ranged from 50% for birth spacing counseling to 59% for smoking screening.

There was variation in the content of care received during the postpartum visit that may have been associated with patient identity. Compared with the privately insured, the Medicaid-insured patients had similar receipt of care with existing standards (78% vs 79%) but higher receipt for other components (33% vs 20%). These findings were similar when comparing White patients with patients of other races and ethnicities, and consistent when examining independent care components (eFigure 1 in Supplement 1). The type of care received was similar among rural and urban residents, except for smoking screening (65% in rural; 58% in urban).

**Figure 2.**
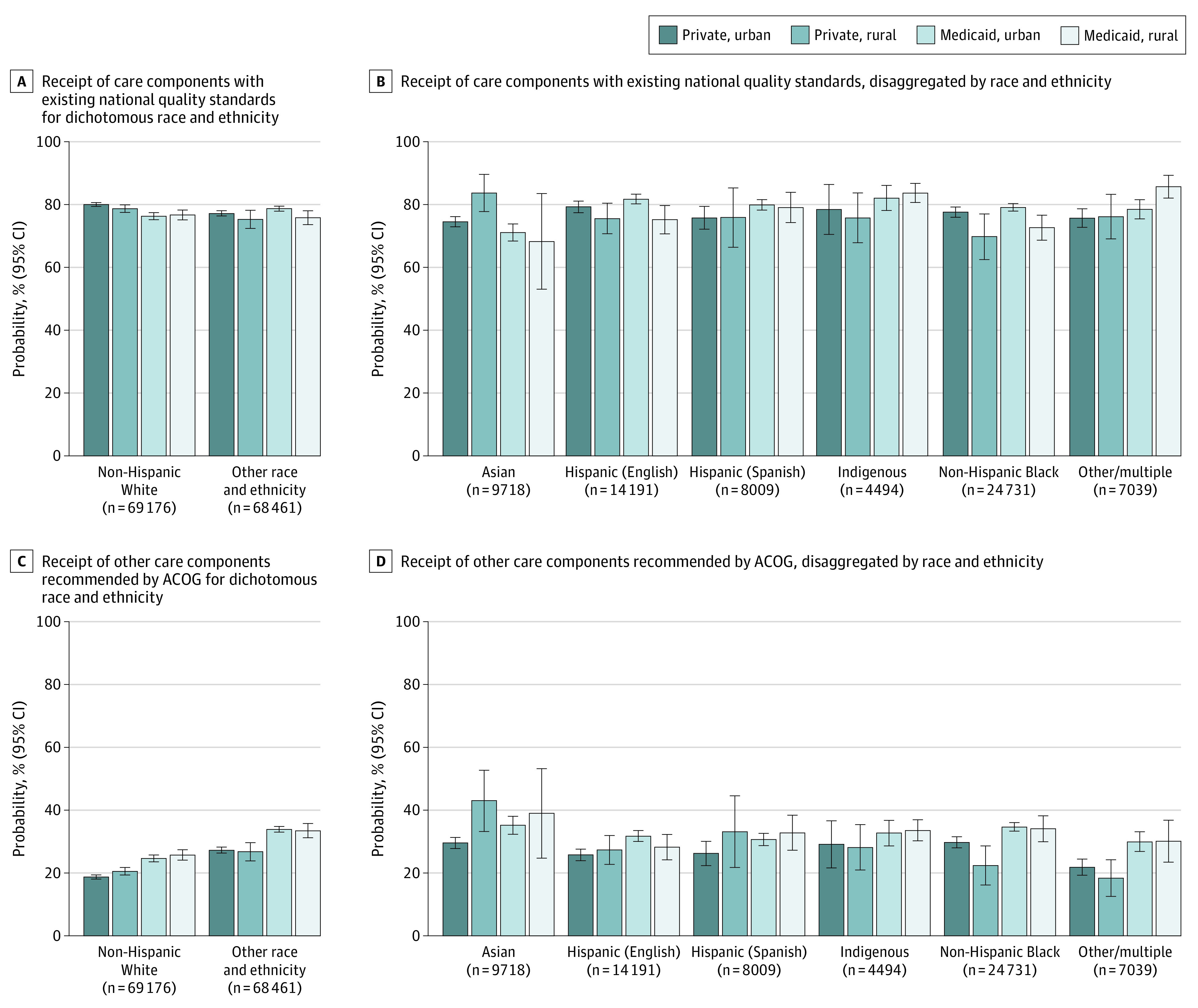
Predicted Probability of Postpartum Care Components at the Intersection of Health Insurance Type, Geography (Rural or Urban), and Race and Ethnicity A, Receipt of care components with existing national quality standards (depression screening and contraceptive counseling) by dichotomous race and ethnicity and B, Disaggregated by other races and ethnicities. C, Receipt of other care components recommended by the ACOG (smoking screening, abuse screening, birth spacing counseling, and discussions about healthy eating and exercise) by dichotomous race and ethnicity and D, Disaggregated by other races and ethnicities. Data were from PRAMS 2016 to 2019 among patients attending a postpartum visit (n = 138 073) and are weighted to account for sample design, nonresponse, and noncoverage. Bars indicate predicted probability with 95% CIs, adjusted for maternal age, education, marital status, parity, pregnancy intention, cesarean delivery, prepregnancy obesity, and prepregnancy and pregnancy smoking, abuse, depression, diabetes, high blood pressure, and hypertension. ACOG refers to the American College of Obstetricians and Gynecologists, and PRAMS, Pregnancy Risk Assessment Monitoring System.

Greater variation in care content was found across intersectional identities. The overall proportion of components received was lowest (59%) among Medicaid-insured White urban residents and highest (64%) among Medicaid-insured, racially minoritized urban residents (eTable 5 in Supplement 1). This variation was largely associated with a higher frequency of other recommended care components (smoking, abuse, birth spacing, eating and exercise) for patients with multiple disadvantaged identities, which persisted after adjustment to risk-standardize across groups (Figure 2). Receipt of these components varied from 19% (95% CI, 18%-19%) among privately insured White urban residents to 35% (95% CI, 33%-36%) among Medicaid-insured Black urban residents and to 43% (95% CI, 33%-53%) among privately insured Asian rural residents.

### Care Components With Existing Standards

**Figure 3.**
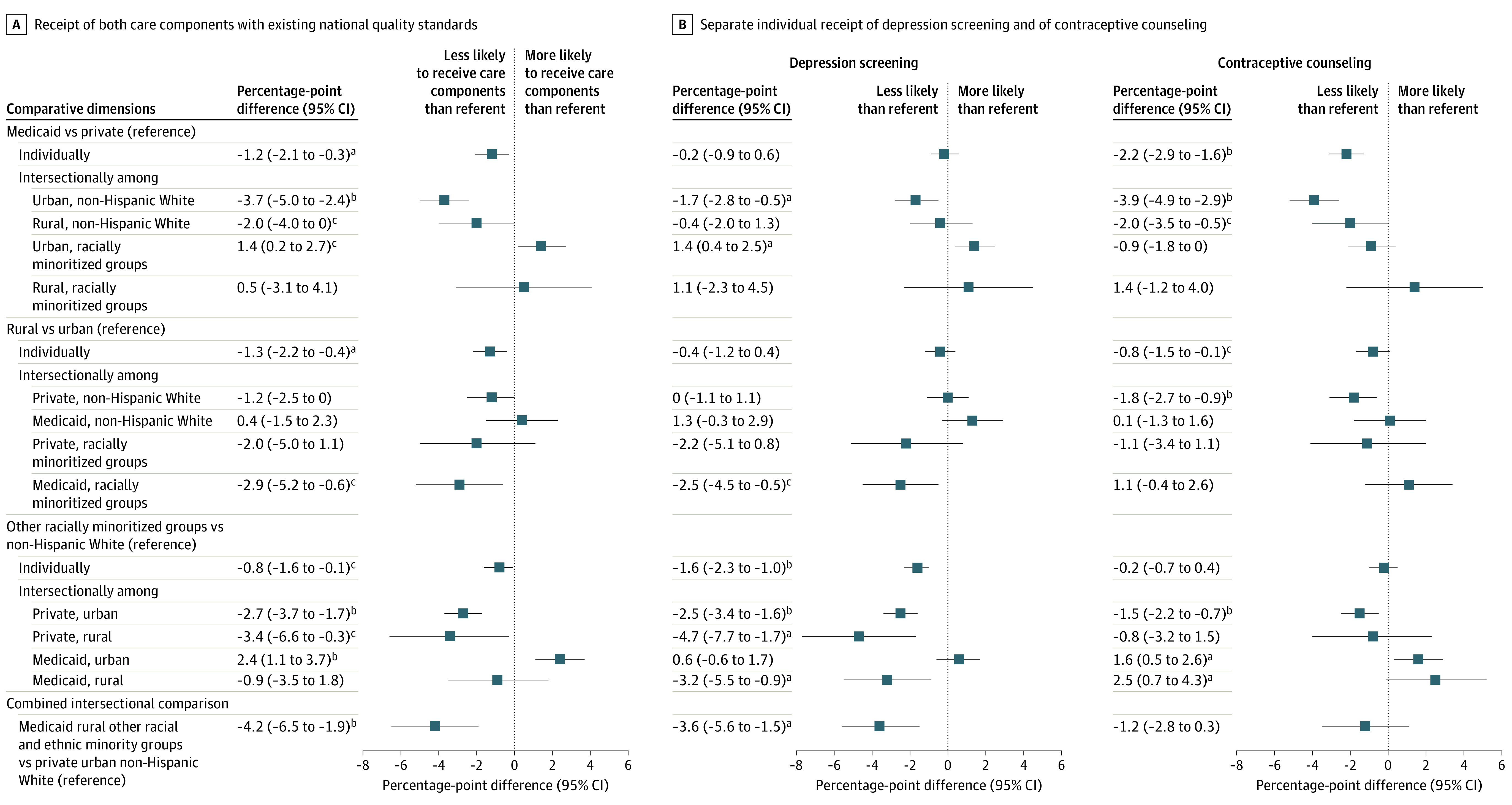
Differences in Receipt of Postpartum Care With Existing National Quality Standards, by Health Insurance Type, Geography (Rural or Urban), and Race and Ethnicity ^a^*P* < .001 ^b^*P* < .01 ^c^*P* < .05 A, Combined receipt of both care components with existing national quality standards. B, Separate, individual receipt of depression screening and of contraceptive counseling. Data are from PRAMS 2016 to 2019 among patients attending a postpartum visit (n = 138 073), and are weighted to account for sample design, nonresponse, and noncoverage. Existing national quality standards include depression screening and contraceptive counseling. Bars show percentage-point risk differences with 95% CIs adjusted for maternal age, education, marital status, parity, pregnancy intention, cesarean delivery, prepregnancy obesity, and prepregnancy and pregnancy smoking, abuse, depression, diabetes, high blood pressure, and hypertension (note that single-identity models also adjust separately for the other individual identifiers). PRAMS refers to Pregnancy Risk Assessment Monitoring System.

When health insurance, geographic residence, and race and ethnicity were examined individually, risk-adjusted differences in care with existing standards (depression, contraception) were all significantly lower for patients insured by Medicaid, rural residents, and people of racially minoritized groups (Figure 3). The Medicaid-private difference (adjusted RD [aRD], –1.2; 95% CI, –1.2 to –0.3) largely reflects the responses of White urban patients (aRD, –3.7; 95% CI, –5.0 to –2.4); and the rural-urban difference (aRD, –1.3; 95% CI, –2.2 to –0.4) reflects Medicaid-insured patients of racially minoritized groups (aRD, –2.9; 95% CI, –5.2 to –0.6). There were fluctuations in the direction of the disparity across race and ethnicity, in which racially minoritized patients were less likely than White patients to receive these components if they were privately insured rural residents (aRD, –3.4; 95% CI, –6.6 to –0.3), but more likely if they were Medicaid-insured urban residents (aRD, 2.4; 95% CI, 1.1 to 3.7).

### Other Recommended Care Components

For other recommended postpartum care components (smoking, abuse, birth spacing, eating/exercise), a different pattern emerged. Risk-adjusted differences were all significantly higher for Medicaid-insured patients (aRD, 6.1; 95% CI, 5.2-7.0), rural residents (aRD, 1.1; 95% CI, 0.1-2.0), and patients of racially minoritized groups (aRD, 8.5; 95% CI, 7.7-9.4; details are available in the eFigure in Supplement 2). Compared with care components with existing national standards, lower proportions of patients received other recommended care components across both individual and intersecting identities, but disparities in care were much greater.

Differences by individual care component and by disaggregated race and ethnicity are further described in eFigures 2 to 3 and eTable 6 in Supplement 1. For example, Medicaid-insured racially minoritized rural residents had a smoking screening rate 17.1 percentage points (95% CI, 14.6-19.6) higher than privately insured White urban residents. This equates to only half (53%) of privately insured White urban residents receiving a smoking screening while almost three-quarters (70%) of their counterparts did so, even after adjustment for smoking status. Findings remained consistent after sensitivity analyses for outcome nonresponse and missing data across site-years (eTables 7 and 8 in Supplement 1).

## Discussion

### Principal Findings

Large differences in postpartum care content exist at the intersection of health insurance, geography, and race and ethnicity, even after adjustment for underlying risk factors. Intersectional differences, which account for cumulative effects of multiple disadvantaged identities,^13^ were consistently much greater than differences between categories in any single identity. Smaller disparities in postpartum care content occurred for care components that have existing national quality standards compared with the other ACOG recommendations examined. However, these components were still received most often by privately insured White urban residents compared with Medicaid-insured racially minoritized rural residents; however, even a small percentage-point difference amounts to many postpartum patients. Receipt of the other recommended care components was extremely low across all patients. Converse to components with existing standards, persistent differences in screenings for topics like smoking and abuse occurred, and these components were received more often by Medicaid-insured racially minoritized rural residents compared with privately insured White urban residents. There is much room for improvement in providing comprehensive postpartum care to all patients and addressing disparities in the receipt of recommended care.

### Findings in the Context of What Is Known

Studies that have examined postpartum care as a binary measure—visit attendance—suggest that postpartum visit attendance is lower among Medicaid-insured compared with privately insured patients^3,17^; these findings are mixed regarding any differences by race and ethnicity. To our knowledge, no studies have examined rural vs urban residence as a variable. Previous studies that have evaluated the content of postpartum visits have focused primarily on depression screening or contraceptive counseling.^32,33,34,35,36,37^ A 2021 study showed that non-Hispanic Black and Hispanic patients received more postpartum screenings than non-Hispanic White patients, as did publicly-insured patients,^36^ findings that are consistent with our own. However, that study did not discuss the role of geography or declining access to care in rural communities,^11^ and did not explore possible associations with intersecting identities. We found that the inclusion of rural residence in our study analyses highlighted the particularly vulnerable circumstances of rural patients who have lower income (ie, Medicaid insured) and are of a racially minoritized group. Prior studies have found that implicit bias, based on socioeconomic status and race and ethnicity, may exist in health screenings.^38,39,40^ Those findings suggest possible mechanisms by which the intersecting identities examined in the present study produced the differences seen in its findings.

### Clinical and Policy Implications

Evaluating postpartum care by recording attendance at a postpartum visit is not sufficient.^17^ Assessing the content and quality of care can underpin clinical and policy efforts for improving the long-term health and well-being of postpartum patients. Furthermore, examining only 1 dimension of identity can mask the range and nature of intersectional risk and disparities in postpartum care. Differences in postpartum care content may be associated with limited time and/or resources or conscious or implicit bias by clinicians or within hospital and clinic policies.^41,42^ The findings of this study show that screenings that have existing national quality standards, which are often tied to financial incentives, are more commonly provided and have smaller differences in screening rates across patient identities. These findings also showed that Medicaid-insured racially minoritized rural residents may lack adequate depression screening and contraceptive counseling, whereas privately insured White urban residents lack adequate postpartum screening for other recommended care, including smoking and abuse. Taken together, these findings indicate that there is a need for universal screening to counteract clinician and policy biases that may affect perception and clinical care for patients with disadvantaged or advantaged identities. The use of universal screening and standardized forms for postpartum care, accompanied by payment incentives, may help address the inequities in care described in this study.^4,17,43^

Expanded use of telehealth may be a way to improve access to postpartum care. However, the benefits of telehealth expansion may accrue differentially across rural and other disadvantaged populations that may have less access to high-speed internet and less privacy for telehealth in the home. Furthermore, continued patient access to telehealth services is contingent on payment reforms that support comprehensive postpartum care and make permanent payment parity policies that pay for telehealth at the same rate as in-person visits.^44,45,46^

### Research Implications

The focus of this analysis was to describe the content of postpartum care across multiple intersecting identities. We used dichotomized measures for patient identities and outcomes of interest to identify potential focal points for clinical and policy efforts. More research is needed to examine individual components and disaggregated racial and ethnic categories, especially among groups with persistently high rates of adverse maternal health outcomes—Black, Indigenous, and Spanish-speaking Hispanic patients, as well as immigrants whose access may be restricted by state Medicaid policies and changes to the Public Charge Rule.^47,48,49,50^ Furthermore, because rural obstetric unit closures are more concentrated in counties where racially minoritized groups are the majority,^51^ future research should ensure that examination of rural geography is included in analyses.

### Limitations

Although PRAMS is a representative sample of postpartum patients (representing 83% of births in the US) and includes assessment of postpartum care content, these data and analyses were subject to limitations. First, the PRAMS data-years used in this study did not include several states (Arizona, California, Idaho, Nevada, Ohio, South Carolina, and Texas), limiting its national generalizability. Second, both social desirability and recall bias may have affected respondents’ answers. However, self-reported surveys provide data that are often impossible to discern from administrative and claims data. Third, PRAMS response rates are higher among White participants compared with participants of other racial or ethnic groups and higher based on socioeconomic advantage, and response bias may interact so that patients who are less likely to receive care are also less likely to participate.^22,52^ Fourth, PRAMS does not ask about all recommended postpartum care components (eg, sleep and fatigue, health maintenance), nor does it include information on the length of time between postpartum visit and survey completion; sterilization (more common among rural residents)^53^ or insertion of long-acting reversible contraception during the childbirth visit (separately reimbursed by many Medicaid programs)^54,55^; health literacy; the quality of counseling provided; or other uncontrolled confounding factors that may affect findings.

## Conclusions

The findings of this cross-sectional survey study suggest that evaluating postpartum care by attendance at a single visit obscures information about the content and quality of care. Inequities in the content of care received are extensive across patient health insurance type, rural or urban residence, and race and ethnicity. Moreover, these disparities are compounded for patients with multiple intersecting disadvantaged identities. Looking at only 1 dimension of identity understates the extent of disparities in postpartum health and care.

## Notes

Supplement 1. eTable 1: Sample size by site and childbirth year among patients included in analyses, PRAMS 2016-2019 eTable 2: ACOG-recommended postpartum care component categories and related questions in PRAMS Phase 8 questionnaire eTable 3: Rates of nonresponse across recommended postpartum care components at the intersection of insurance, geography, race and ethnicity, PRAMS 2016-2019 eTable 4: Participant characteristics by postpartum visit attendance, PRAMS 2016-2019 eTable 5: Participant characteristics at the intersection of insurance, geography, race and ethnicity, PRAMS 2016-2019 eFigure 1: Weighted proportion of patients who received recommended postpartum care by care component, insurance, geography, race and ethnicity, PRAMS 2016-2019 (n=153,683) eFigure 2: Unadjusted and adjusted predicted probability of recommended postpartum care by individual care component, insurance, geography, and disaggregated race and ethnicity, PRAMS 2016-2019 eFigure 3: Adjusted percentage-point differences in receipt of recommended postpartum care components among patients attending a postpartum visit, PRAMS 2016-2019 eTable 6: Adjusted percentage-point differences in receipt of recommended postpartum care components by insurance, geography, race and ethnicity (separately and at their intersections) among patients attending a postpartum visit, PRAMS 2016-2019 eTable 7: Sensitivity analyses for treatment of missing values in main analyses, PRAMS 2016-2019 eTable 8: Sensitivity analyses for missing state-years in main analyses, PRAMS 2016-2019

Supplement 2. eFigure: Receipt of Other Recommended Care Components
