# Supplementary material for: Association of Health Insurance, Geography, and Race and Ethnicity With Disparities in Receipt of Recommended Postpartum Care in the US
Source: JAMA Health Forum. 2022 Oct 14;3(10):e223292. doi: 10.1001/jamahealthforum.2022.3292 (PMC9568809; doi:10.1001/jamahealthforum.2022.3292)
Supplement: Supplement 1. — eTable 1: Sample size by site and childbirth year among patients included in analyses, PRAMS 2016-2019 eTable 2: ACOG-recommended postpartum care component categories and related questions in PRAMS Phase 8 questionnaire eTable 3: Rates of nonresponse across recommended postpartum care components at the intersection of insurance, geography, race and ethnicity, PRAMS 2016-2019 eTable 4: Participant characteristics by postpartum visit attendance, PRAMS 2016-2019 eTable 5: Participant characteristics at the intersection of insurance, geography, race and ethnicity, PRAMS 2016-2019 eFigure 1: Weighted proportion of patients who received recommended postpartum care by care component, insurance, geography, race and ethnicity, PRAMS 2016-2019 (n=153,683) eFigure 2: Unadjusted and adjusted predicted probability of recommended postpartum care by individual care component, insurance, geography, and disaggregated race and ethnicity, PRAMS 2016-2019 eFigure 3: Adjusted percentage-point differences in receipt of recommended postpartum care components among patients attending a postpartum visit, PRAMS 2016-2019 eTable 6: Adjusted percentage-point differences in receipt of recommended postpartum care components by insurance, geography, race and ethnicity (separately and at their intersections) among patients attending a postpartum visit, PRAMS 2016-2019 eTable 7: Sensitivity analyses for treatment of missing values in main analyses, PRAMS 2016-2019 eTable 8: Sensitivity analyses for missing state-years in main analyses, PRAMS 2016-2019 [file jamahealthforum-e223292-s001.pdf]

# Supplemental Online Content

Interrante JD, Admon LK, Carroll C, Henning-Smith C, Chastain P, Kozhimannil KB. Association of health insurance, geography, and race and ethnicity with disparities in receipt of recommended postpartum care in the US. *JAMA Health Forum.* 2022;3(10):e223292. doi:10.1001/jamahealthforum.2022.3292

**eTable 1**: Sample size by site and childbirth year among patients included in analyses, PRAMS 2016-2019

**eTable 2**: ACOG-recommended postpartum care component categories and related questions in PRAMS Phase 8 questionnaire

**eTable 3**: Rates of nonresponse across recommended postpartum care components at the intersection of insurance, geography, race and ethnicity, PRAMS 2016-2019

**eTable 4**: Participant characteristics by postpartum visit attendance, PRAMS 2016-2019

**eTable 5**: Participant characteristics at the intersection of insurance, geography, race and ethnicity, PRAMS 2016-2019

**eFigure 1**: Weighted proportion of patients who received recommended postpartum care by care component, insurance, geography, race and ethnicity, PRAMS 2016-2019 (n=153,683)

**eFigure 2**: Unadjusted and adjusted predicted probability of recommended postpartum care by individual care component, insurance, geography, and disaggregated race and ethnicity, PRAMS 2016-2019

**eFigure 3**: Adjusted percentage-point differences in receipt of recommended postpartum care components among patients attending a postpartum visit, PRAMS 2016-2019

**eTable 6**: Adjusted percentage-point differences in receipt of recommended postpartum care components by insurance, geography, race and ethnicity (separately and at their intersections) among patients attending a postpartum visit, PRAMS 2016-2019

**eTable 7**: Sensitivity analyses for treatment of missing values in main analyses, PRAMS 2016-2019

**eTable 8**: Sensitivity analyses for missing state-years in main analyses, PRAMS 2016-2019

This supplemental material has been provided by the authors to give readers additional information about their work.

© 2022 Interrante JD et al. *JAMA Health Forum.*

**eTable 1: Sample size by site and childbirth year among patients included in analyses, PRAMS 2016-2019**

| | Sample Size by Childbirth Year (N) | | | |
|---|---|---|---|---|
| **PRAMS Site** | **2016** | **2017** | **2018** | **2019** |
| Alabama | — | 862 | 795 | 781 |
| Alaska | 1076 | 970 | 914 | 965 |
| Arizona | — | — | — | — |
| Arkansas | 872 | — | 875 | 952 |
| California | — | — | — | — |
| Colorado | 1586 | 1098 | 1076 | 1053 |
| Connecticut | 1224 | 1354 | 1344 | 1124 |
| District of Columbia | — | — | 516 | 513 |
| Delaware | 923 | 876 | 826 | 909 |
| Florida | — | — | — | 1009 |
| Georgia | — | 906 | 702 | 754 |
| Hawaii | 1047 | — | — | 613 |
| Idaho | — | — | — | — |
| Illinois | 1358 | 1212 | 1291 | 1220 |
| Indiana | — | — | 815 | — |
| Iowa | 932 | 972 | 804 | 799 |
| Kansas | — | 948 | 916 | 946 |
| Kentucky | — | 679 | 731 | 874 |
| Louisiana | 879 | 892 | 853 | 1069 |
| Maine | 789 | 853 | 803 | 795 |
| Maryland | 1116 | 1023 | 999 | 931 |
| Massachusetts | 1305 | 1411 | 1425 | 1689 |
| Michigan | 1787 | 1886 | 1860 | 1382 |
| Minnesota | — | — | 1243 | 1039 |
| Mississippi | — | — | 1179 | 1233 |
| Missouri | 1026 | 1076 | 906 | 1566 |
| Montana | — | 872 | 762 | 658 |
| Nebraska | 1262 | — | 1271 | 1549 |
| Nevada | — | — | — | — |
| New Hampshire | 561 | 576 | 574 | 652 |
| New Jersey | 1225 | 1152 | 1103 | 1034 |
| New Mexico | 1080 | 1087 | 1091 | 1077 |
| New York City | 1429 | 1286 | 1533 | 1268 |
| New York, Other | 937 | 835 | 758 | 752 |
| North Carolina | — | 917 | 857 | 794 |
| North Dakota | — | 544 | 820 | 704 |
| Ohio | — | — | — | — |
| Oklahoma | 1798 | 1581 | 1360 | — |
| Oregon | — | — | 1584 | 2407 |

© 2022 Interrante JD et al. *JAMA Health Forum.*

| | | | | |
|---|---|---|---|---|
| Pennsylvania | 1005 | 1180 | 917 | 1104 |
| Rhode Island | 1140 | 1093 | 1095 | 988 |
| South Carolina | — | — | — | — |
| South Dakota | — | 1068 | 916 | 937 |
| Tennessee | — | — | — | 660 |
| Texas | — | — | — | — |
| Utah | 1316 | 1350 | 1184 | 1620 |
| Vermont | 910 | 837 | 859 | 771 |
| Virginia | 639 | 977 | 1038 | 899 |
| Washington | 1266 | 1229 | 1128 | 1167 |
| West Virginia | 784 | 688 | 674 | — |
| Wisconsin | 1249 | 1330 | 1041 | 832 |
| Wyoming | 571 | 474 | 509 | 461 |

PRAMS: Pregnancy Risk Assessment Monitoring System.

Analyses were restricted to patients with either Medicaid or private insurance, which account for 97% of all births in the sample. Sites that do not participate in PRAMS did not contribute data for any study year. For all other sites, in the years 2016 and 2017, PRAMS required a minimum overall response rate threshold of 55% in order to release site data. The threshold was lowered to 50% for 2018 and 2019.

© 2022 Interrante JD et al. *JAMA Health Forum.*

**eTable 2: ACOG-recommended postpartum care component categories and related questions in PRAMS Phase 8 questionnaire**

| ACOG Category | Component | PRAMS Questions | Analytic Category |
|---|---|---|---|
| Mood and emotional well-being | Depression screen | *During your postpartum checkup or postpartum home visit, did a doctor, nurse, or other health care worker ask if you were feeling down or depressed?* | *Existing national quality standard* |
| | Smoking screen | *During your postpartum checkup or postpartum home visit, did a doctor, nurse, or other health care worker ask you if you were smoking cigarettes?* | *Other recommended* |
| | Abuse screening | *During your postpartum checkup or postpartum home visit, did a doctor, nurse, or other health care worker ask if someone was hurting you emotionally or physically?* | *Other recommended* |
| Sexuality, contraception, and birth spacing | Contraceptive counseling | *During your postpartum checkup or postpartum home visit, did a doctor, nurse, or other health care worker talk to you about birth control methods you can use after giving birth?* | *Existing national quality standard* |
| | | *During your postpartum checkup, did a doctor, nurse, or other health care worker give or prescribe you a contraceptive method such as the pill, patch, shot (Depo-Provera®), NuvaRing®, or condoms?* | |
| | | *During your postpartum checkup, did a doctor, nurse, or other health care worker insert an IUD (Mirena®, ParaGard®, Liletta®, or Skyla®) or a contraceptive implant (Nexplanon® or Implanon®)?* | |
| | Birth spacing | *During your postpartum checkup or postpartum home visit, did a doctor, nurse, or other health care worker talk to you about how long to wait before getting pregnant again?* | *Other recommended* |
| Sleep and fatigue | | Not asked | |
| Physical recovery from birth | Healthy eating/ exercise | *During your postpartum checkup or postpartum home visit, did a doctor, nurse, or other health care worker talk to you about healthy eating, exercise, and losing weight gained during pregnancy?* | *Other recommended* |
| Health maintenance | | Not asked | |

ACOG: The American College of Obstetricians and Gynecologists; PRAMS: Pregnancy Risk Assessment Monitoring System.

© 2022 Interrante JD et al. *JAMA Health Forum.*

**eTable 3: Rates of nonresponse across recommended postpartum care components at the intersection of insurance, geography, race and ethnicity, PRAMS 2016-2019**

| | Unweighted N (%) | | | | | | | | |
|---|---|---|---|---|---|---|---|---|---|
| **Categories** | | **Existing National Quality Standards** | | | **Other recommended by ACOG** | | | | |
| | **Postpartum visit** | **Depression Screen** | **Contraceptive Counseling** | **Both** | **Smoking Screen** | **Abuse Screen** | **Birth Spacing** | **Eating/ Exercise** | **All** |
| Overall | 2274 (1.5) | 1946 (1.4) | 1551 (1.1) | 1513 (1.1) | 2637 (1.9) | 2382 (1.7) | 2420 (1.8) | 2068 (1.5) | 1837 (1.3) |
| | | | | | | | | | |
| By intersectional group | | | | | | | | | |
| Private-urban-non-Hispanic white | 230 (0.6) | 234 (0.6) | 172 (0.4) | 160 (0.4) | 398 (1.0) | 327 (0.8) | 273 (0.7) | 238 (0.6) | 258 (0.7) |
| Private-rural-non-Hispanic white | 64 (0.6) | 76 (0.7) | 63 (0.6) | 60 (0.6) | 117 (1.1) | 93 (0.9) | 88 (0.8) | 74 (0.7) | 73 (0.7) |
| Private-urban-other race/ethnicities | 332 (1.3) | 356 (1.4) | 262 (1.1) | 253 (1.0) | 534 (2.2) | 445 (1.8) | 473 (1.9) | 354 (1.4) | 299 (1.2) |
| Private-rural-other race/ethnicities | 44 (1.3) | 30 (1.0) | 25 (0.8) | 23 (0.8) | 43 (1.4) | 41 (1.3) | 41 (1.3) | 38 (1.2) | 34 (1.1) |
| Medicaid-urban-non-Hispanic white | 205 (1.5) | 214 (1.8) | 175 (1.5) | 180 (1.5) | 253 (2.1) | 265 (2.2) | 243 (2.0) | 228 (1.9) | 222 (1.8) |
| Medicaid-rural-non-Hispanic white | 81 (1.0) | 103 (1.5) | 83 (1.2) | 86 (1.3) | 126 (1.8) | 118 (1.7) | 111 (1.6) | 99 (1.5) | 100 (1.5) |
| Medicaid-urban-other race/ethnicities | 198 (2.2) | 783 (2.3) | 650 (1.9) | 634 (1.9) | 988 (3.0) | 902 (2.7) | 1018 (3.0) | 871 (2.6) | 706 (2.1) |
| Medicaid-rural-other race/ethnicities | 12 (2.1) | 140 (2.0) | 112 (1.6) | 109 (1.5) | 166 (2.3) | 175 (2.4) | 163 (2.3) | 158 (2.2) | 137 (1.9) |
| | | | | | | | | | |
| Missing insurance, rural/urban residence, or race/ethnicity | 12 (2.1) | 10 (2.1) | 9 (1.9) | 8 (1.7) | 12 (2.5) | 16 (3.4) | 10 (2.1) | 8 (1.7) | 8 (1.7) |

ACOG: The American College of Obstetricians and Gynecologists; PRAMS: Pregnancy Risk Assessment Monitoring System.

Care components were restricted to those reporting a postpartum visit. Insurance was missing for 9 participants, rural/urban residence in 39, race/ethnicity in 512, and any insurance, geography or race/ethnicity in 560.

© 2022 Interrante JD et al. *JAMA Health Forum.*

**eTable 4: Participant characteristics by postpartum visit attendance, PRAMS 2016-2019**

| | Postpartum visit | |
|---|---|---|
| | **Yes** | **No/Unknown** |
| | **(n=138,073)** | **(n=15,610)** |
| **Characteristic** | **% (95% CI)** | **% (95% CI)** |
| **Identity dimensions** | | |
| Insurance status | | |
| Private | 94.8 (94.6,95.0) | 5.2 (5.0,5.4) |
| Medicaid | 85.2 (84.8,85.7) | 14.8 (14.3,15.2) |
| Geographic residence | | |
| Urban | 90.8 (90.6,91.1) | 9.2 (8.9,9.4) |
| Rural | 89.9 (89.3,90.4) | 10.1 (9.6,10.7) |
| Race and ethnicity | | |
| Non-Hispanic white | 93.0 (92.7,93.2) | 7.0 (6.8,7.3) |
| Other race and ethnicities | 87.4 (87.0,87.8) | 12.6 (12.2,13.0) |
| Non-Hispanic Black | 86.5 (85.8,87.2) | 13.5 (12.8,14.2) |
| Hispanic (English-speaking) | 88.3 (87.5,89.2) | 11.7 (10.8,12.5) |
| Hispanic (Spanish-speaking) | 85.6 (84.5,86.7) | 14.4 (13.3,15.5) |
| Indigenous | 80.1 (78.4,81.9) | 19.9 (18.1,21.6) |
| Asian | 91.4 (90.5,92.2) | 8.6 (7.8,9.5) |
| Other/multiple | 87.9 (86.5,89.3) | 12.1 (10.7,13.5) |
| **Other sociodemographics** | | |
| Age | | |
| <20 | 83.6 (82.1,85.1) | 16.4 (14.9,17.9) |
| 20-24 | 86.8 (86.2,87.5) | 13.2 (12.5,13.8) |
| 25-34 | 91.7 (91.4,92.0) | 8.3 (8.0,8.6) |
| ≥35 | 92.9 (92.5,93.4) | 7.1 (6.6,7.5) |
| Education | | |
| Less than high school | 80.8 (79.8,81.8) | 19.2 (18.2,20.2) |
| High school | 86.5 (85.9,87.1) | 13.5 (12.9,14.1) |
| More than high school | 94.0 (93.7,94.2) | 6.0 (5.8,6.3) |
| Marital status | | |
| Not married | 85.9 (85.4,86.3) | 14.1 (13.7,14.6) |
| Married | 93.6 (93.4,93.9) | 6.4 (6.1,6.6) |
| **Other health characteristics** | | |
| Parity | | |
| Primiparous | 92.8 (92.5,93.1) | 7.2 (6.9,7.5) |
| Multiparous | 89.3 (89.0,89.6) | 10.7 (10.4,11.0) |
| Delivery type | | |
| Vaginal | 90.1 (89.8,90.4) | 9.9 (9.6,10.2) |
| Cesarean | 91.8 (91.5,92.2) | 8.2 (7.8,8.5) |
| Pregnancy intention | | |
| Intended | 93.0 (92.8,93.3) | 7.0 (6.7,7.2) |

© 2022 Interrante JD et al. *JAMA Health Forum.*

| | | |
|---|---|---|
| Unintended | 87.5 (87.1,87.9) | 12.5 (12.1,12.9) |
| Pre-pregnancy obesity | | |
| No | 90.8 (90.5,91.0) | 9.2 (9.0,9.5) |
| Yes | 90.6 (90.1,91.0) | 9.4 (9.0,9.9) |
| Pre-pregnancy or pregnancy | | |
| Smoking | | |
| No | 91.9 (91.7,92.1) | 8.1 (7.9,8.3) |
| Yes | 84.9 (84.2,85.6) | 15.1 (14.4,15.8) |
| Physical abuse | | |
| No | 91.8 (91.5,92.0) | 8.2 (8.0,8.5) |
| Yes | 84.2 (82.9,85.6) | 15.8 (14.4,17.1) |
| Depression | | |
| No | 91.4 (91.1,91.6) | 8.6 (8.4,8.9) |
| Yes | 87.6 (87.0,88.3) | 12.4 (11.7,13.0) |
| Diabetes | | |
| No | 90.6 (90.3,90.8) | 9.4 (9.2,9.7) |
| Yes | 91.6 (91.0,92.2) | 8.4 (7.8,9.0) |
| High blood pressure/ hypertension | | |
| No | 90.6 (90.4,90.9) | 9.4 (9.1,9.6) |
| Yes | 91.1 (90.5,91.6) | 8.9 (8.4,9.5) |

PRAMS: Pregnancy Risk Assessment Monitoring System.

Data are weighted to account for sample design, nonresponse, and noncoverage.

© 2022 Interrante JD et al. *JAMA Health Forum.*

**eTable 5: Participant characteristics at the intersection of insurance, geography, race and ethnicity, PRAMS 2016-2019**

| | | Privately Insured | | | | Medicaid Insured | | | |
|---|---|---|---|---|---|---|---|---|---|
| | | Non-Hispanic White | | Other Race and Ethnicities | | Non-Hispanic White | | Other Race and Ethnicities | |
| | All | Urban | Rural | Urban | Rural | Urban | Rural | Urban | Rural |
| | (n=153,683) | (n=41,284) | (n=11,156) | (n=26,652) | (n=3,341) | (n=14,029) | (n=7,890) | (n=39,777) | (n=8,994) |
| **Characteristic** | **% (95% CI)** | **% (95% CI)** | **% (95% CI)** | **% (95% CI)** | **% (95% CI)** | **% (95% CI)** | **% (95% CI)** | **% (95% CI)** | **% (95% CI)** |
| Postpartum visit | 90.7 (90.4,90.9) | 96.0 (95.7,96.2) | 94.4 (93.7,95.1) | 92.5 (92.0,93.1) | 91.6 (89.8,93.4) | 86.1 (85.3,87.0) | 87.7 (86.6,88.8) | 84.4 (83.8,85.0) | 83.0 (81.5,84.6) |
| Mean proportion of care components received | 61.0 (60.8,61.3) | 59.0 (58.7,59.4) | 60.3 (59.4,61.2) | 62.9 (62.3,63.5) | 61.9 (59.6,64.1) | 58.8 (58.0,59.7) | 61.2 (60.0,62.4) | 64.1 (63.5,64.7) | 62.9 (61.4,64.5) |
| **Among patients attending a postpartum visit (n)** | **138,073** | **39,632** | **10,581** | **24,761** | **3,059** | **12,096** | **6,843** | **33,432** | **7,192** |
| **Sociodemographics** | | | | | | | | | |
| Age | | | | | | | | | |
| <20 | 3.9 (3.7,4.0) | 0.9 (0.8,1.0) | 1.8 (1.4,2.3) | 1.8 (1.5,2.1) | 3.3 (2.0,4.6) | 6.5 (5.8,7.2) | 10.7 (9.6,11.9) | 7.4 (6.9,7.9) | 10.4 (9.0,11.7) |
| 20-24 | 17.6 (17.3,17.9) | 8.5 (8.1,8.9) | 17.7 (16.5,19.0) | 12.3 (11.6,13.1) | 21.2 (18.2,24.1) | 27.5 (26.4,28.7) | 33.3 (31.6,35.1) | 25.9 (25.1,26.7) | 32.7 (30.4,35.0) |
| 25-34 | 59.5 (59.1,59.9) | 66.5 (65.8,67.1) | 66.2 (64.8,67.6) | 59.8 (58.7,60.8) | 57.2 (53.8,60.6) | 54.6 (53.3,55.8) | 47.5 (45.7,49.4) | 52.1 (51.2,53.0) | 48.5 (46.1,50.9) |
| ≥35 | 19.0 (18.7,19.4) | 24.1 (23.5,24.7) | 14.3 (13.3,15.3) | 26.1 (25.2,27.0) | 18.3 (15.6,21.1) | 11.4 (10.6,12.2) | 8.4 (7.4,9.4) | 14.6 (14.0,15.2) | 8.5 (7.3,9.7) |
| Education | | | | | | | | | |
| Less than high school | 9.6 (9.3,9.8) | 1.0 (0.9,1.2) | 2.5 (2.0,3.0) | 4.2 (3.7,4.6) | 6.9 (5.4,8.5) | 14.2 (13.3,15.2) | 16.8 (15.4,18.3) | 24.8 (24.0,25.6) | 24.9 (22.8,27.0) |
| High school | 23.2 (22.9,23.6) | 10.3 (9.9,10.8) | 17.4 (16.1,18.6) | 16.3 (15.5,17.1) | 25.1 (22.1,28.1) | 38.6 (37.4,39.9) | 44.3 (42.4,46.1) | 36.6 (35.8,37.5) | 41.9 (39.5,44.3) |
| More than high school | 66.4 (66.0,66.8) | 88.3 (87.8,88.7) | 80.0 (78.7,81.3) | 78.9 (78.0,79.8) | 67.7 (64.5,70.8) | 46.4 (45.2,47.7) | 38.6 (36.8,40.4) | 37.5 (36.6,38.3) | 32.1 (29.9,34.3) |
| Married | 63.9 (63.5,64.3) | 87.9 (87.4,88.4) | 82.8 (81.5,84.1) | 71.3 (70.3,72.3) | 64.6 (61.3,68.0) | 42.2 (41.0,43.5) | 38.8 (37.0,40.6) | 32.8 (31.9,33.6) | 29.4 (27.3,31.6) |
| **Other health characteristics** | | | | | | | | | |
| Multiparous | 60.0 (59.6,60.4) | 55.5 (54.8,56.2) | 59.3 (57.8,60.7) | 55.8 (54.8,56.9) | 61.2 (57.9,64.4) | 64.4 (63.2,65.7) | 65.6 (63.8,67.3) | 66.3 (65.5,67.2) | 67.9 (65.7,70.1) |
| Cesarean delivery | 33.5 (33.2,33.9) | 32.4 (31.8,33.0) | 32.7 (31.3,34.1) | 37.7 (36.7,38.7) | 35.1 (31.9,38.2) | 31.8 (30.6,33.0) | 33.0 (31.3,34.7) | 33.9 (33.0,34.7) | 34.2 (32.0,36.5) |
| Unintended pregnancy | 38.8 (38.4,39.2) | 23.9 (23.3,24.5) | 28.0 (26.6,29.3) | 38.1 (37.1,39.1) | 43.8 (40.5,47.2) | 51.0 (49.7,52.2) | 54.3 (52.4,56.1) | 55.9 (55.0,56.8) | 57.4 (55.0,59.8) |
| Pre-pregnancy obesity | 29.9 (29.6,30.3) | 23.9 (23.3,24.5) | 33.0 (31.6,34.4) | 28.9 (28.0,29.9) | 38.1 (34.7,41.4) | 32.5 (31.3,33.7) | 38.0 (36.2,39.8) | 35.2 (34.4,36.1) | 41.5 (39.1,43.9) |
| Pre-pregnancy or pregnancy | | | | | | | | | |
| Smoking | 16.3 (16.0,16.6) | 10.5 (10.1,11.0) | 15.2 (14.1,16.4) | 6.6 (6.1,7.1) | 10.9 (9.0,12.9) | 37.6 (36.4,38.8) | 44.5 (42.7,46.4) | 14.3 (13.7,14.9) | 23.0 (21.1,24.9) |
| Physical abuse | 4.1 (3.9,4.2) | 1.8 (1.6,2.0) | 2.1 (1.7,2.5) | 2.9 (2.6,3.3) | 3.4 (2.4,4.5) | 7.4 (6.7,8.0) | 10.0 (8.8,11.2) | 5.7 (5.3,6.1) | 8.2 (7.0,9.5) |
| Depression | 17.7 (17.4,18.0) | 14.1 (13.7,14.6) | 17.9 (16.6,19.1) | 11.7 (11.0,12.3) | 14.8 (12.5,17.1) | 30.2 (29.1,31.4) | 35.9 (34.1,37.7) | 16.7 (16.1,17.4) | 18.9 (17.2,20.6) |
| Diabetes | 12.0 (11.7,12.2) | 10.7 (10.3,11.1) | 11.6 (10.6,12.6) | 14.0 (13.3,14.7) | 14.3 (12.1,16.6) | 11.3 (10.5,12.2) | 11.5 (10.3,12.8) | 13.2 (12.6,13.8) | 12.6 (11.2,14.1) |
| High blood pressure | 16.3 (16.0,16.6) | 15.4 (14.9,15.8) | 17.9 (16.7,19.1) | 14.7 (14.0,15.5) | 17.1 (14.5,19.7) | 17.4 (16.4,18.4) | 19.6 (18.1,21.1) | 16.7 (16.1,17.4) | 18.8 (17.0,20.6) |

PRAMS: Pregnancy Risk Assessment Monitoring System.

Data are weighted to account for sample design, nonresponse, and noncoverage. Sum of column percentages may not equal 100% because of missing data. Missing one of the three identities in n=560 overall (n=477 among patients attending a postpartum visit).

© 2022 Interrante JD et al. *JAMA Health Forum.*

**eFigure 1: Weighted proportion of patients who received recommended postpartum care by care component, insurance, geography, race and ethnicity, PRAMS 2016-2019 (n=153,683)**

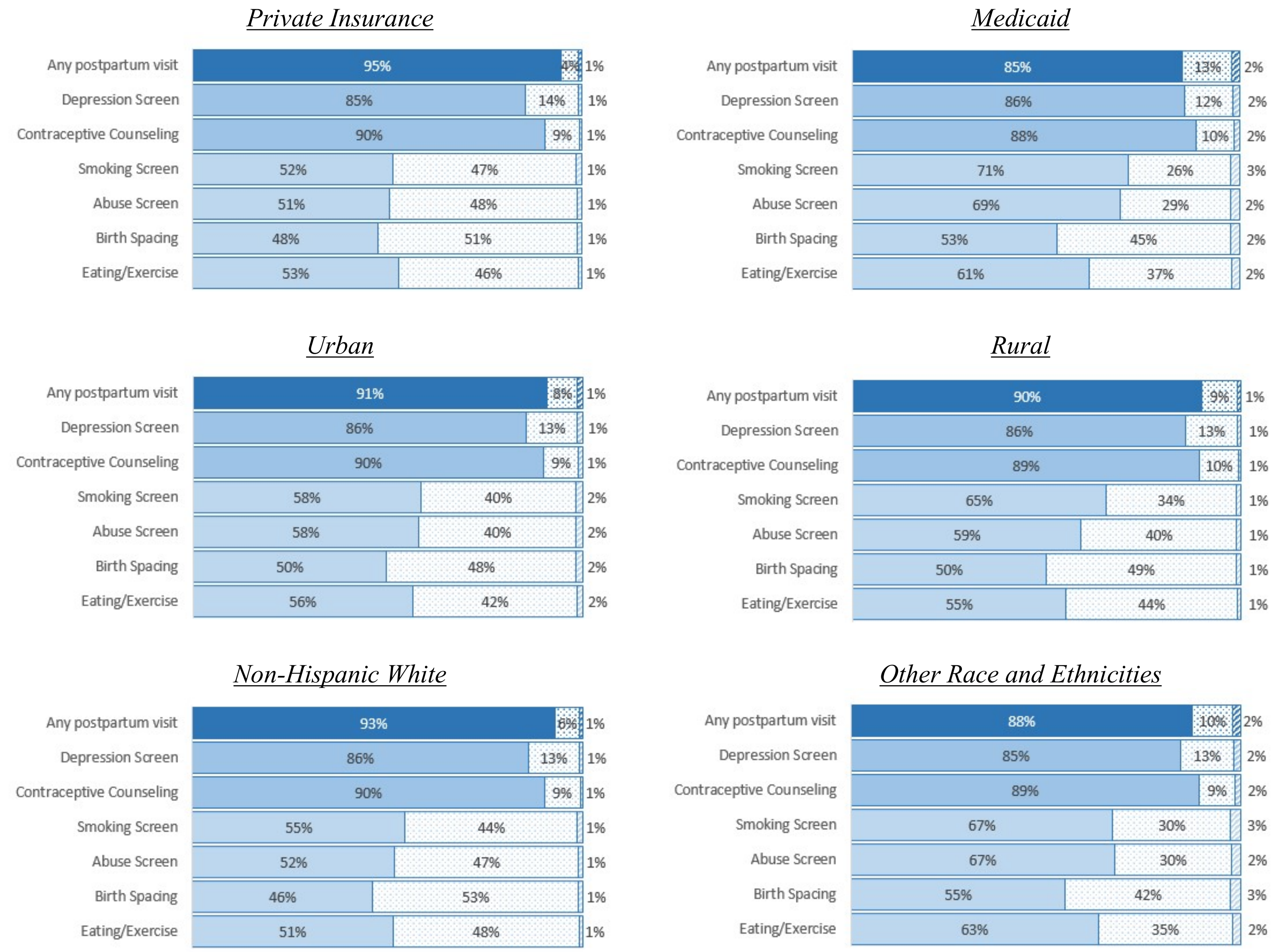


PRAMS: Pregnancy Risk Assessment Monitoring System.

Data are weighted to account for sample design, nonresponse, and noncoverage. Solid bars indicate receipt, dotted bars indicate no receipt, and diagonal stripped bars indicate unknown. Percentages within specific care components are restricted those patients attending a postpartum visit.

© 2022 Interrante JD et al. *JAMA Health Forum.*

**eFigure 2: Unadjusted and adjusted predicted probability of recommended postpartum care by individual care component, insurance, geography, and disaggregated race and ethnicity, PRAMS 2016-2019**

<u>A. Existing National Quality Standards</u>

a.1. Depression screen - unadjusted

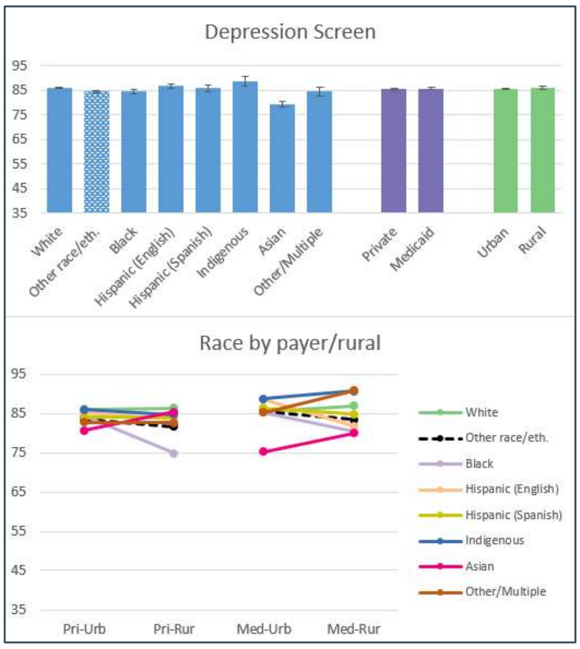


a.2. Depression screen – adjusted

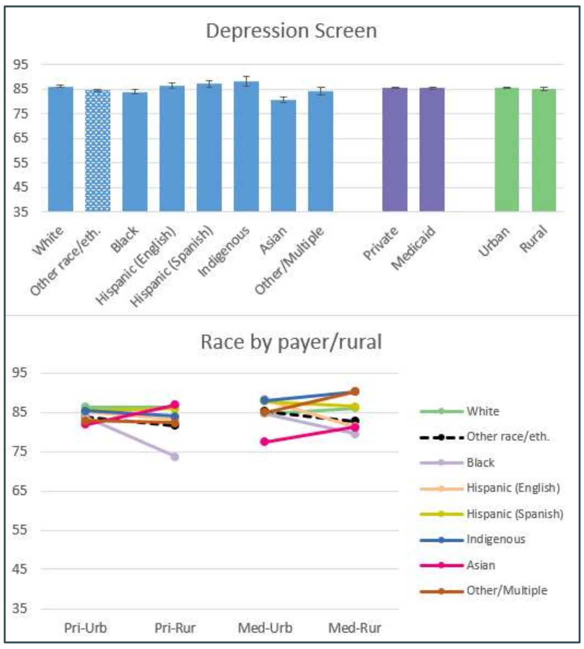


b.1. Contraceptive counseling – unadjusted

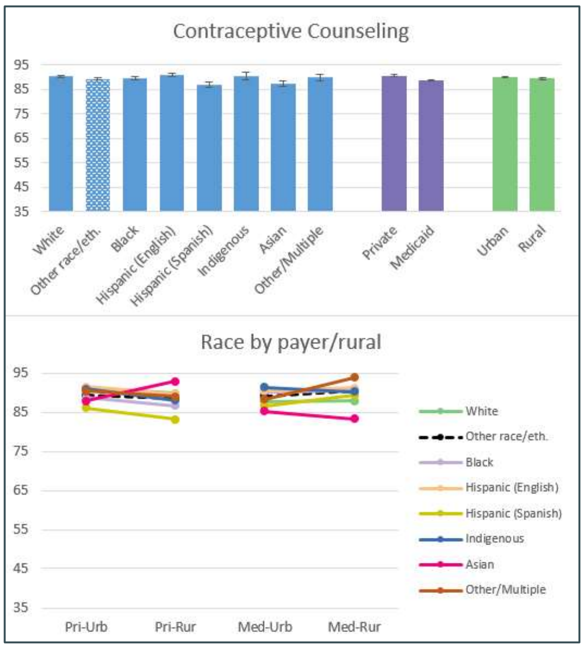


b.2. Contraceptive counseling – adjusted

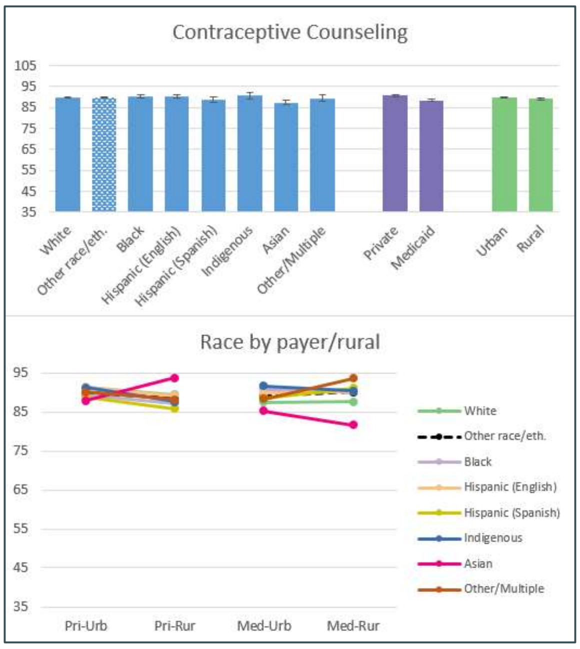


© 2022 Interrante JD et al. *JAMA Health Forum.*

B. Other Care Components Recommended by ACOG

a.1. Smoking screening – unadjusted

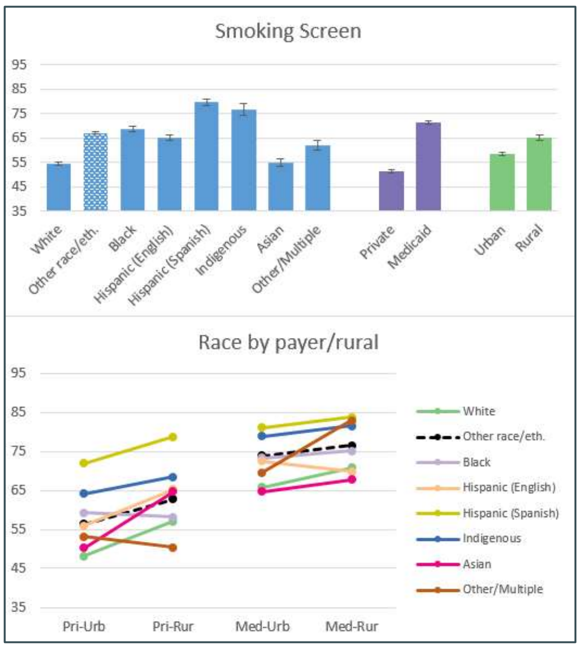


a.2. Smoking screening – adjusted

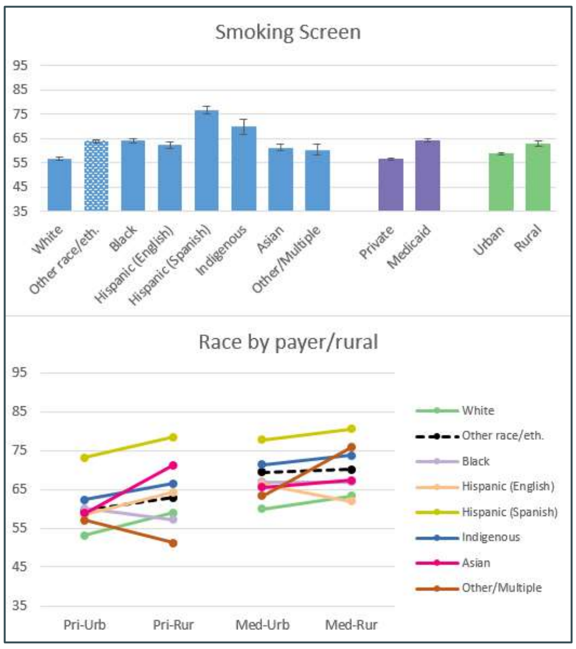


b.1. Abuse screen – unadjusted

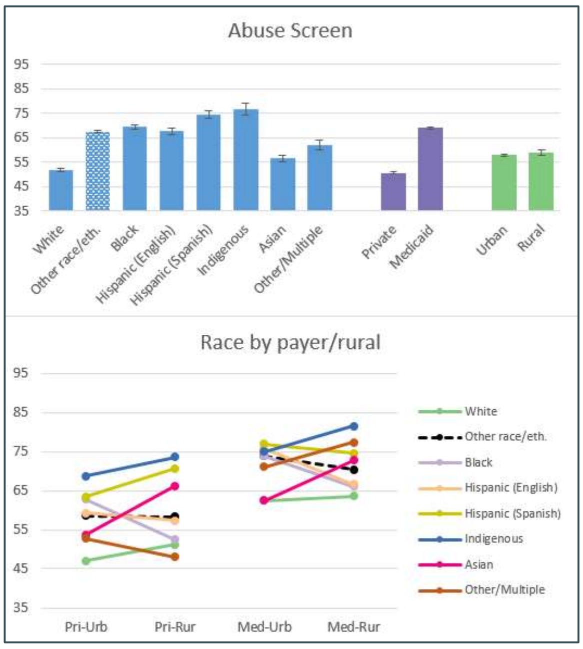


b.2. Abuse screen – adjusted

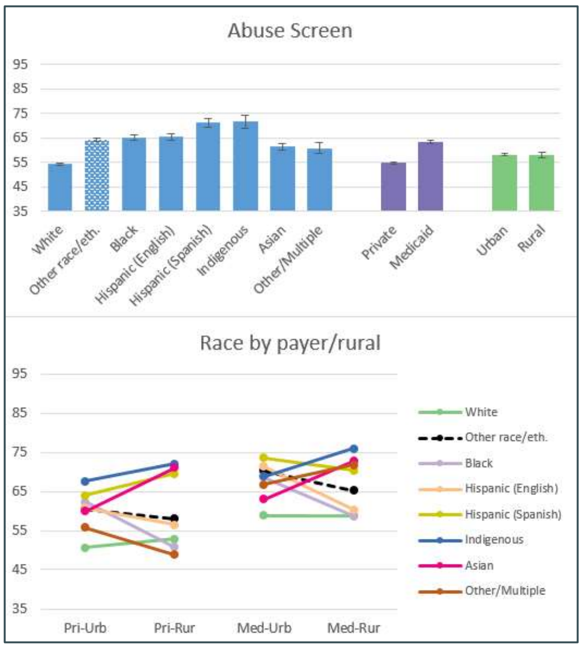


c.1. Birth spacing – unadjusted

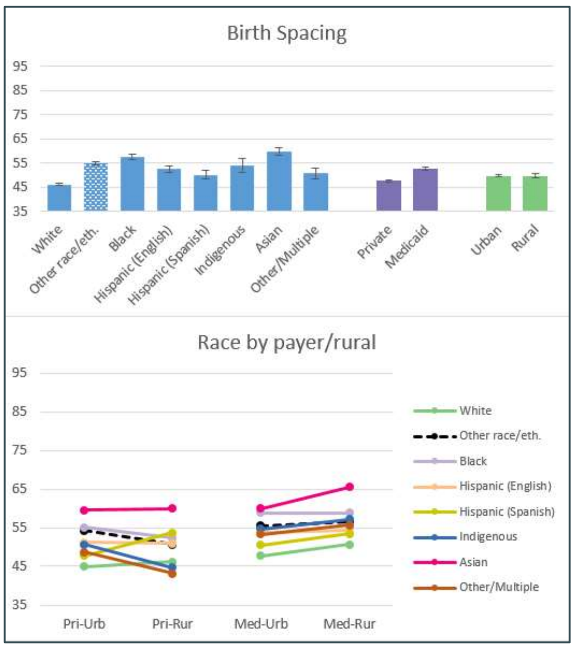


c.2. Birth spacing – adjusted

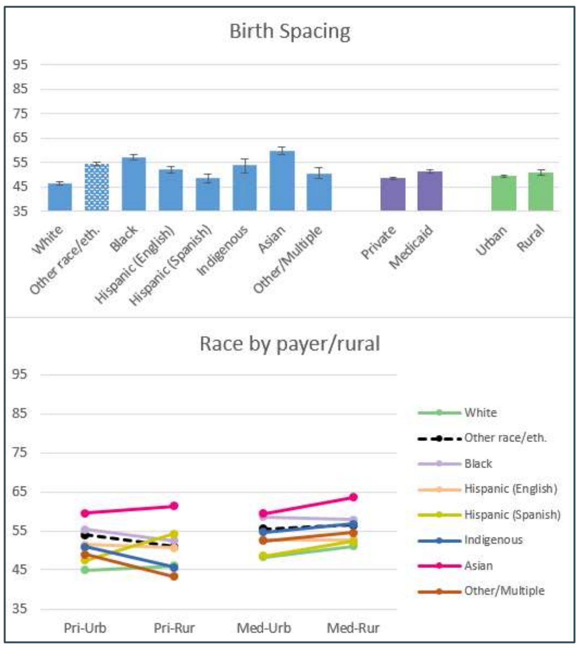


© 2022 Interrante JD et al. *JAMA Health Forum.*

d.1. Eating/exercise – unadjusted

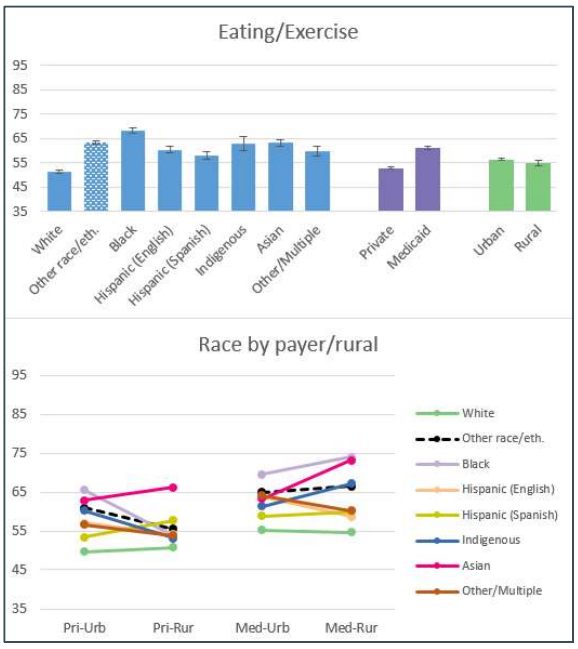


d.2. Eating/exercise – adjusted

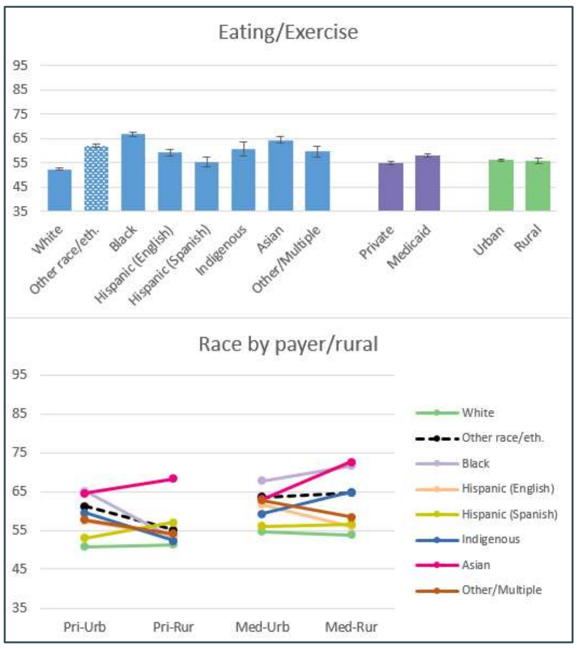


ACOG: The American College of Obstetricians and Gynecologists; PRAMS: Pregnancy Risk Assessment Monitoring System.

Top bars show differences across individual dimensions; bottom lines show rural/urban differences by payer for each racial and ethnicity category. Data are weighted to account for sample design, nonresponse, and noncoverage. Adjusted results control for maternal age, education, marital status, parity, pregnancy intension, cesarean delivery, pre-pregnancy obesity, and pre-pregnancy and pregnancy smoking, abuse, depression, diabetes, high blood pressure, and hypertension (Note: Single-dimension models also adjust separately for the other individual identifiers).

**eFigure 3: Adjusted percentage-point differences in receipt of recommended postpartum care components among patients attending a postpartum visit, PRAMS 2016-2019**

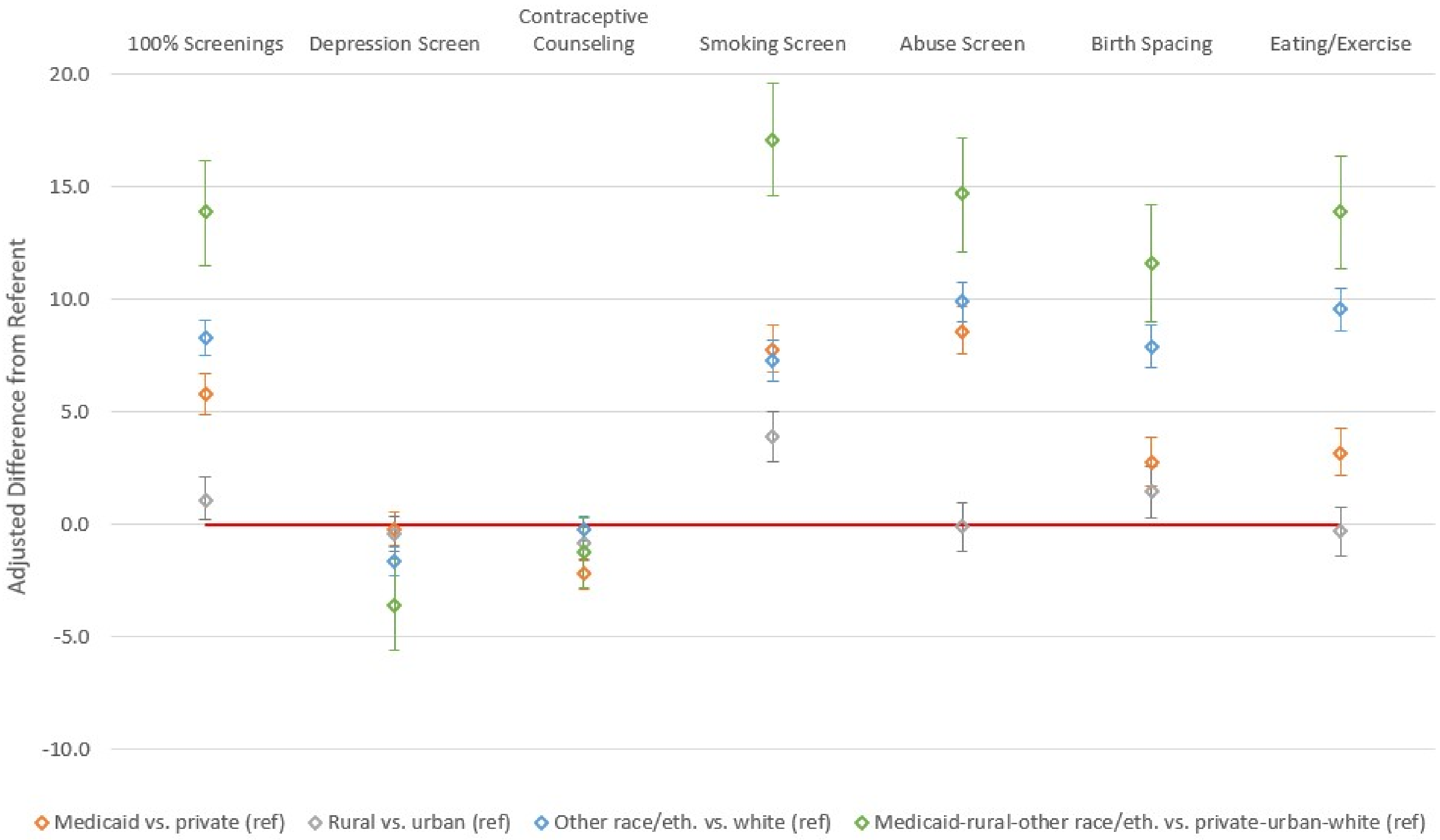


PRAMS: Pregnancy Risk Assessment Monitoring System.

Data are weighted to account for sample design, nonresponse, and noncoverage. Adjusted for maternal age, education, marital status, parity, pregnancy intention, cesarean delivery, pre-pregnancy obesity, and pre-pregnancy and pregnancy smoking, abuse, depression, diabetes, high blood pressure, and hypertension (single-identity model is also adjusted for the other individual identifiers).

© 2022 Interrante JD et al. *JAMA Health Forum.*

**eTable 6: Adjusted percentage-point differences in receipt of recommended postpartum care components by insurance, geography, race and ethnicity (separately and at their intersections) among patients attending a postpartum visit, PRAMS 2016-2019**

| | Existing national quality standards | | | | | | Other ACOG recommended components | | | | | | | | | | | |
|---|---|---|---|---|---|---|---|---|---|---|---|---|---|---|---|---|---|---|
| | Depression Screening | | | Contraception Counseling | | | Smoking Screening | | | Abuse Screening | | | Birth Spacing | | | Eating/Exercise | | |
| | % | 95% CI | | % | 95% CI | | % | 95% CI | | % | 95% CI | | % | 95% CI | | % | 95% CI | |
| **Single identity percentage-point differences** | | | | | | | | | | | | | | | | | | |
| Medicaid vs. private (ref) | -0.2 | -0.9,0.6 | | -2.2 | -2.9,-1.6 | *** | 7.8 | 6.8,8.9 | *** | 8.6 | 7.6,9.7 | *** | 2.8 | 1.7,3.9 | *** | 3.2 | 2.2,4.3 | *** |
| Rural vs. urban (ref) | -0.4 | -1.2,0.4 | | -0.8 | -1.5,-0.1 | * | 3.9 | 2.8,5.0 | *** | -0.1 | -1.2,1.0 | | 1.5 | 0.3,2.6 | * | -0.3 | -1.4,0.8 | |
| Other race and ethnicities vs. non-Hispanic white (ref) | -1.6 | -2.3,-1.0 | *** | -0.2 | -0.7,0.4 | | 7.3 | 6.4,8.2 | *** | 9.9 | 9.0,10.8 | *** | 7.9 | 7.0,8.9 | *** | 9.6 | 8.6,10.5 | *** |
| | | | | | | | | | | | | | | | | | | |
| **Intersectional percentage-point differences** | | | | | | | | | | | | | | | | | | |
| Medicaid vs. private (ref) among: | | | | | | | | | | | | | | | | | | |
| Urban, non-Hispanic white | -1.7 | -2.8,-0.5 | ** | -3.9 | -4.9,-2.9 | *** | 6.9 | 5.4,8.5 | *** | 8.3 | 6.7,9.8 | *** | 3.2 | 1.7,4.8 | *** | 3.9 | 2.3,5.5 | *** |
| Rural, non-Hispanic white | -0.4 | -2.0,1.3 | | -2.0 | -3.5,-0.5 | * | 4.3 | 1.9,6.7 | ** | 5.9 | 3.5,8.4 | *** | 5.0 | 2.5,7.4 | *** | 2.5 | 0.0,5.0 | * |
| Urban, other race and ethnicities | 1.4 | 0.4,2.5 | ** | -0.9 | -1.8,0.0 | | 9.8 | 8.4,11.2 | *** | 9.9 | 8.5,11.3 | *** | 1.6 | 0.1,3.0 | * | 2.4 | 1.0,3.8 | ** |
| Rural, other race and ethnicities | 1.1 | -2.3,4.5 | | 1.4 | -1.2,4.0 | | 7.3 | 3.3,11.3 | *** | 7.2 | 3.1,11.4 | ** | 5.6 | 1.4,9.7 | ** | 9.6 | 5.5,13.7 | *** |
| Rural vs. urban (ref) among: | | | | | | | | | | | | | | | | | | |
| Private, non-Hispanic white | 0.0 | -1.1,1.1 | | -1.8 | -2.7,-0.9 | *** | 5.9 | 4.3,7.4 | *** | 2.2 | 0.6,3.8 | ** | 1.2 | -0.4,2.8 | | 0.5 | -1.1,2.1 | |
| Medicaid, non-Hispanic white | 1.3 | -0.3,2.9 | | 0.1 | -1.3,1.6 | | 3.2 | 1.0,5.4 | ** | -0.1 | -2.3,2.1 | | 3.0 | 0.7,5.2 | * | -0.9 | -3.2,1.4 | |
| Private, other race and ethnicities | -2.2 | -5.1,0.8 | | -1.1 | -3.4,1.1 | | 3.3 | 0.0,6.7 | | -2.4 | -6.0,1.1 | | -3.0 | -6.6,0.5 | | -6.1 | -9.6,-2.5 | ** |
| Medicaid, other race and ethnicities | -2.5 | -4.5,-0.5 | * | 1.1 | -0.4,2.6 | | 0.8 | -1.6,3.3 | | -5.1 | -7.5,-2.6 | *** | 1.0 | -1.5,3.5 | | 1.1 | -1.3,3.5 | |
| Other race and ethnicities vs. non-Hispanic white (ref) among: | | | | | | | | | | | | | | | | | | |
| Private, urban | -2.5 | -3.4,-1.6 | *** | -1.5 | -2.2,-0.7 | *** | 6.5 | 5.3,7.7 | *** | 9.8 | 8.6,11.1 | *** | 9.0 | 7.8,10.3 | *** | 10.4 | 9.2,11.6 | *** |
| Private, rural | -4.7 | -7.7,-1.7 | ** | -0.8 | -3.2,1.5 | | 3.9 | 0.4,7.5 | * | 5.2 | 1.5,8.9 | ** | 4.8 | 1.1,8.5 | * | 3.8 | 0.1,7.5 | * |
| Medicaid, urban | 0.6 | -0.6,1.7 | | 1.6 | 0.5,2.6 | ** | 9.4 | 7.8,10.9 | *** | 11.4 | 9.9,13.0 | *** | 7.4 | 5.8,9.0 | *** | 8.9 | 7.3,10.5 | *** |

© 2022 Interrante JD et al. *JAMA Health Forum.*

| | | | | | | | | | | | | | | | | | | |
|---|---|---|---|---|---|---|---|---|---|---|---|---|---|---|---|---|---|---|
| Medicaid, rural | -3.2 | -5.5,-0.9 | ** | 2.5 | 0.7,4.3 | ** | 7.0 | 4.0,9.9 | *** | 6.5 | 3.5,9.5 | *** | 5.4 | 2.4,8.4 | *** | 10.9 | 8.0,13.8 | *** |
| Medicaid-rural-other race/ethnicities vs. private-urban-non-Hispanic white | -3.6 | -5.6,-1.5 | ** | -1.2 | -2.8,0.3 | | 17.1 | 14.6,19.6 | *** | 14.7 | 12.1,17.2 | *** | 11.6 | 9.0,14.2 | *** | 13.9 | 11.4,16.4 | *** |

* $p < 0.05$, ** $p < 0.01$, *** $p < 0.001$.

PRAMS: Pregnancy Risk Assessment Monitoring System.

Data are weighted to account for sample design, nonresponse, and noncoverage. Adjusted for maternal age, education, marital status, parity, pregnancy intention, cesarean delivery, pre-pregnancy obesity, and pre-pregnancy and pregnancy smoking, abuse, depression, diabetes, high blood pressure, and hypertension (single-identity model is also adjusted for the other individual identifiers).

© 2022 Interrante JD et al. *JAMA Health Forum.*

**eTable 7: Sensitivity analyses for treatment of missing values in main analyses, PRAMS 2016-2019**

| | **Existing National Quality Standards** | | | | | | | **Other Recommended by ACOG** | | | | | |
|---|---|---|---|---|---|---|---|---|---|---|---|---|---|
| | Percentage-Point Difference (95% CI) | | | | | | | Percentage-Point Difference (95% CI) | | | | | |
| **Comparisons** | **Main Analysis (n=138,073)** | | **Complete Outcome (n=136,088)** | | **MICE (n=138,073)** | | | **Main Analysis (n=138,073)** | | **Complete Outcome (n=136,088)** | | **MICE (n=138,073)** | |
| **Medicaid vs. private (ref)** | | | | | | | | | | | | | |
| Individual | -1.2 (-2.1,-0.3) | ** | -0.7 (-1.6,0.2) | | -0.8 (-1.6,0.1) | | | 6.1 (5.2,7.0) | ** | 6.4 (5.4,7.3) | *** | 6.3 (5.4,7.3) | *** |
| Intersectional among: | | | | | | | | | | | | | |
| Urban, non-Hispanic white | -3.7 (-5.0,-2.4) | *** | -3.2 (-4.5,-1.9) | *** | -3.3 (-4.6,-2.0) | *** | | 6.0 (4.7,7.3) | *** | 6.1 (4.8,7.5) | *** | 6.1 (4.8,7.4) | *** |
| Rural, non-Hispanic white | -2.0 (-4.0,0.0) | * | -1.8 (-3.7,0.2) | | -2.0 (-3.9,0.0) | | | 5.2 (3.1,7.2) | *** | 5.3 (3.2,7.4) | *** | 5.2 (3.2,7.3) | *** |
| Urban, other race and ethnicities | 1.4 (0.2,2.7) | * | 2.2 (1.0,3.4) | *** | 2.1 (0.9,3.3) | ** | | 6.6 (5.2,8.0) | *** | 7.0 (5.6,8.4) | *** | 6.9 (5.6,8.3) | *** |
| Rural, other race and ethnicities | 0.5 (-3.1,4.1) | | -0.3 (-3.8,3.2) | | -1.8 (-0.4,0.6) | | | 6.7 (3.1,10.4) | *** | 6.6 (2.9,10.3) | ** | 6.3 (3.8,8.9) | *** |
| **Rural vs. urban (ref)** | | | | | | | | | | | | | |
| Individual | -1.3 (-2.2,-0.4) | ** | -1.5 (-2.4,-0.6) | ** | -1.5 (-2.5,-0.6) | ** | | 1.1 (0.1,2.0) | ** | 1.0 (-0.0,2.0) | | 1.0 (-0.0,2.0) | |
| Intersectional among: | | | | | | | | | | | | | |
| Private, non-Hispanic white | -1.2 (-2.5,0.0) | | -1.3 (-2.6,0.0) | * | -1.2 (-2.5,0.1) | | | 1.9 (0.5,3.2) | ** | 1.8 (0.5,3.2) | ** | 1.8 (0.4,3.2) | ** |
| Medicaid, non-Hispanic white | 0.4 (-1.5,2.3) | | 0.2 (-1.7,2.1) | | 0.1 (-1.7,2.0) | | | 1.0 (-0.9,3.0) | | 1.0 (-1.0,2.9) | | 1.0 (-1.0,2.9) | |
| Private, other race and ethnicities | -2.0 (-5.0,1.1) | | -1.3 (-4.2,1.6) | | -1.6 (-4.5,1.4) | | | -0.5 (-3.5,2.5) | | -0.3 (-3.3,2.8) | | -0.2 (-3.4,2.9) | |
| Medicaid, other race and ethnicities | -2.9 (-5.2,-0.6) | * | -3.8 (-6.1,-1.5) | ** | -3.9 (-6.1,-1.6) | ** | | -0.4 (-2.7,2.0) | | -0.7 (-3.1,1.7) | | -0.6 (-3.0,1.8) | |
| **Other race and ethnicities vs. non-Hispanic white (ref)** | | | | | | | | | | | | | |
| Individual | -0.8 (-1.6,-0.1) | * | -0.4 (-1.2,0.4) | | -0.4 (-1.2,0.3) | | | 8.5 (7.7,9.4) | * | 8.8 (8.0,9.7) | *** | 8.9 (8.0,9.6) | *** |
| Intersectional among: | | | | | | | | | | | | | |
| Private, urban | -2.7 (-3.7,-1.7) | *** | -2.4 (-3.4,-1.3) | *** | -2.4 (-3.4,-1.3) | *** | | 8.6 (7.5,9.6) | *** | 8.8 (7.7,9.8) | *** | 8.7 (7.6,9.8) | *** |
| Private, rural | -3.4 (-6.6,-0.3) | * | -2.4 (-5.4,0.6) | | -2.7 (-5.8,0.4) | | | 6.2 (3.1,9.3) | *** | 6.6 (3.5,9.8) | *** | 6.7 (3.4,9.9) | *** |
| Medicaid, urban | 2.4 (1.1,3.7) | *** | 3.1 (1.7,4.4) | *** | 3.0 (1.7,4.4) | *** | | 9.2 (7.8,10.6) | *** | 9.6 (8.2,11.1) | *** | 9.6 (8.2,11.0) | *** |
| Medicaid, rural | -0.9 (-3.5,1.8) | | -0.9 (-3.6,1.7) | | -1.0 (-3.6,1.7) | | | 7.8 (5.0,10.5) | *** | 7.9 (5.1,10.7) | *** | 8.0 (5.2,10.8) | *** |
| **Combined intersectional comparison** | | | | | | | | | | | | | |
| Medicaid-rural-other race and ethnicities vs. private-urban-non-Hispanic white (ref) | -4.2 (-6.5,-1.9) | *** | -4.0 (-6.3,-1.6) | ** | -4.2 (-6.5,-1.8) | *** | | 14.8 (12.4,17.2) | *** | 15.1 (12.6,17.5) | *** | 15.1 (12.6,17.5) | *** |

* $p < 0.05$, ** $p < 0.01$, *** $p < 0.001$

© 2022 Interrante JD et al. *JAMA Health Forum.*

ACOG: The American College of Obstetricians and Gynecologists; PRAMS: Pregnancy Risk Assessment Monitoring System.

Complete outcome column uses listwise deletion for missing outcome variables. MICE column uses imputation on outcomes by multiple imputation by chained equations (MICE) with m=10 and all covariates from the main model included. Adjusted for maternal age, education, marital status, parity, pregnancy intension, cesarean delivery, pre-pregnancy obesity, and pre-pregnancy and pregnancy smoking, abuse, depression, diabetes, high blood pressure, and hypertension (Note: Individual comparison models also adjust separately for the other individual identifiers). Existing national quality standards include depression screening and contraception counseling. Other care components recommended by the ACOG include smoking screening, abuse screening, birth spacing counseling, and discussions about healthy eating and exercise.

© 2022 Interrante JD et al. *JAMA Health Forum.*

**eTable 8: Sensitivity analyses for missing state-years in main analyses, PRAMS 2016-2019**

| | **Existing National Quality Standards** | | | | | | | | **Other Recommended by ACOG** | | | | | | | |
|---|---|---|---|---|---|---|---|---|---|---|---|---|---|---|---|---|
| | Percentage-Point Difference (95% CI) | | | | | | | | Percentage-Point Difference (95% CI) | | | | | | | |
| **Comparisons** | **Main Analysis (n=138,073)** | | **State Clustered Standard Errors (n=138,073)** | | **States With All Years Available (n=96,139)** | | **2018-2019 Only (n=76,036)** | | **Main Analysis (n=138,073)** | | **State Clustered Standard Errors (n=138,073)** | | **States With All Years Available (n=96,139)** | | **2018-2019 Only (n=76,036)** | |
| **Medicaid vs. private (ref)** | | | | | | | | | | | | | | | | |
| Individual | -1.2 (-2.1,-0.3) | ** | -1.2 (-2.6,0.2) | | -2.0 (-3.0,-1.0) | *** | -0.8 (-2.0,0.4) | | 6.1 (5.2,7.0) | ** | 6.1 (5.1,7.1) | *** | 5.4 (4.3,6.4) | *** | 6.2 (4.9,7.4) | *** |
| Intersectional among: | | | | | | | | | | | | | | | | |
| Urban, non-Hispanic white | -3.7 (-5.0,-2.4) | *** | -3.7 (-6.3,-1.1) | ** | -4.7 (-6.3,-3.2) | *** | -2.8 (-4.6,-1.0) | ** | 6.0 (4.7,7.3) | *** | 6.0 (4.9,7.0) | *** | 4.9 (3.4,6.4) | *** | 6.9 (5.1,8.8) | *** |
| Rural, non-Hispanic white | -2.0 (-4.0,0.0) | * | -2.0 (-4.3,0.2) | | -3.2 (-5.8,-0.7) | * | -2.0 (-4.6,0.6) | | 5.2 (3.1,7.2) | *** | 5.2 (3.1,7.2) | *** | 3.9 (1.2,6.6) | ** | 4.3 (1.5,7.1) | ** |
| Urban, other race and ethnicities | 1.4 (0.2,2.7) | * | 1.4 (-0.1,2.9) | | 1.2 (-0.2,2.5) | | 1.3 (-0.4,3.0) | | 6.6 (5.2,8.0) | *** | 6.6 (4.8,8.3) | *** | 6.1 (4.6,7.6) | *** | 6.1 (4.2,8.0) | *** |
| Rural, other race and ethnicities | 0.5 (-3.1,4.1) | | 0.5 (-4.6,5.6) | | -2.3 (-7.0,2.4) | | 1.4 (-3.6,6.4) | | 6.7 (3.1,10.4) | *** | 6.7 (3.0,10.5) | ** | 7.8 (2.7,12.8) | ** | 6.4 (1.4,11.5) | * |
| **Rural vs. urban (ref)** | | | | | | | | | | | | | | | | |
| Individual | -1.3 (-2.2,-0.4) | ** | -1.3 (-3.7,1.1) | | 0.5 (-0.7,1.6) | | -1.4 (-2.7,-0.2) | * | 1.1 (0.1,2.0) | ** | 1.1 (-0.3,2.4) | | 1.9 (0.6,3.2) | ** | 1.5 (0.2,2.8) | * |
| Intersectional among: | | | | | | | | | | | | | | | | |
| Private, non-Hispanic white | -1.2 (-2.5,0.0) | | -1.2 (-3.9,1.4) | | 0.5 (-1.0,2.1) | | -1.0 (-2.6,0.7) | | 1.9 (0.5,3.2) | ** | 1.9 (0.0,3.7) | | 2.5 (0.7,4.2) | ** | 2.9 (1.0,4.7) | ** |
| Medicaid, non-Hispanic white | 0.4 (-1.5,2.3) | | 0.4 (-3.0,3.8) | | 2.1 (-0.3,4.5) | | -0.2 (-2.7,2.3) | | 1.0 (-0.9,3.0) | | 1.0 (-0.7,2.8) | | 1.5 (-0.9,3.9) | | 0.2 (-2.4,2.9) | |
| Private, other race and ethnicities | -2.0 (-5.0,1.1) | | -2.0 (-6.3,2.3) | | 1.7 (-2.2,5.5) | | -3.2 (-7.5,1.0) | | -0.5 (-3.5,2.5) | | -0.5 (-3.6,2.6) | | -0.1 (-4.2,4.0) | | 0.2 (-3.9,4.4) | |
| Medicaid, other race and ethnicities | -2.9 (-5.2,-0.6) | * | -2.9 (-6.5,0.8) | | -1.8 (-4.7,1.2) | | -3.1 (-6.0,-0.1) | * | -0.4 (-2.7,2.0) | | -0.4 (-2.6,1.9) | | 1.6 (-1.6,4.7) | | 0.5 (-2.7,3.8) | |
| **Other race and ethnicities vs. non-Hispanic white (ref)** | | | | | | | | | | | | | | | | |
| Individual | -0.8 (-1.6,-0.1) | * | -0.8 (-2.0,0.3) | | -1.1 (-2.0,-0.2) | * | -0.5 (-1.5,0.6) | | 8.5 (7.7,9.4) | * | 8.5 (7.6,9.5) | *** | 8.5 (7.6,9.5) | *** | 9.0 (7.9,10.1) | *** |
| Intersectional among: | | | | | | | | | | | | | | | | |
| Private, urban | -2.7 (-3.7,-1.7) | *** | -2.7 (-4.5,-0.9) | ** | -3.3 (-4.4,-2.2) | *** | -1.9 (-3.3,-0.5) | * | 8.6 (7.5,9.6) | *** | 8.6 (7.1,10.0) | *** | 8.1 (7.0,9.3) | *** | 9.5 (8.0,11.0) | *** |
| Private, rural | -3.4 (-6.6,-0.3) | * | -3.4 (-7.2,0.3) | | -2.2 (-6.2,1.8) | | -4.1 (-8.5,0.2) | | 6.2 (3.1,9.3) | *** | 6.2 (3.3,9.1) | *** | 5.6 (1.3,9.9) | * | 6.9 (2.6,11.2) | ** |
| Medicaid, urban | 2.4 (1.1,3.7) | *** | 2.4 (0.5,4.3) | * | 2.6 (1.1,4.2) | ** | 2.2 (0.4,4.0) | * | 9.2 (7.8,10.6) | *** | 9.2 (7.8,10.6) | *** | 9.3 (7.8,10.9) | *** | 8.7 (6.7,10.7) | *** |
| Medicaid, rural | -0.9 (-3.5,1.8) | | -0.9 (-4.4,2.7) | | -1.2 (-4.7,2.3) | | -0.7 (-4.1,2.8) | | 7.8 (5.0,10.5) | *** | 7.8 (5.5,10.1) | *** | 9.4 (5.7,13.2) | *** | 9.0 (5.3,12.8) | *** |
| **Combined intersectional comparison** | | | | | | | | | | | | | | | | |
| Medicaid-rural-other race and ethnicities vs. private-urban-non-Hispanic white (ref) | -4.2 (-6.5,-1.9) | *** | -4.2 (-8.1,-0.3) | * | -3.9 (-6.9,-0.9) | * | -3.7 (-6.7,-0.6) | * | 14.8 (12.4,17.2) | *** | 14.8 (12.4,17.2) | *** | 15.8 (12.6,19.0) | *** | 16.2 (12.9,19.5) | *** |

* $p < 0.05$, ** $p < 0.01$, *** $p < 0.001$

ACOG: The American College of Obstetricians and Gynecologists; PRAMS: Pregnancy Risk Assessment Monitoring System.

"State clustering" column takes the conservative approach by only including state-level clustering rather than the precision benefit of the survey stratum (each state in PRAMS has their own stratified sampling scheme, which is accounted for by a stratum variable with values unique for stratums within each state-year; this stratum variable is used in all main analyses along with PRAMS sampling weights; to the best of our knowledge, there is no way to both account for the PRAMS sampling stratum and simultaneously have higher-level (state) clustering, as the standard errors are already clustered at the strata-level). "States with all years available" column uses includes those states with PRAMS data available for every year from 2016 through 2019: Alaska, Colorado, Connecticut, Delaware, Illinois, Iowa, Louisiana, Maine, Maryland, Massachusetts, Michigan, Missouri, New Hampshire, New Jersey, New Mexico, New York City, New York State, Pennsylvania, Rhode Island, Utah, Vermont, Virginia, Washington, Wisconsin, Wyoming. The "2018-2019 only" column restricts analyses to those years where the response rate threshold for data release was lowered from 55% to 50%, meaning that more states met the inclusion criteria for data release. Adjusted for maternal age, education, marital status, parity, pregnancy intension, cesarean delivery, pre-pregnancy obesity, and pre-pregnancy and pregnancy smoking, abuse, depression, diabetes, high blood pressure, and hypertension (Note: Individual comparison models also adjust separately for the other individual identifiers). Existing national quality standards include depression screening and contraception counseling. Other care components recommended by the ACOG include smoking screening, abuse screening, birth spacing counseling, and discussions about healthy eating and exercise.

© 2022 Interrante JD et al. *JAMA Health Forum.*
